# A low-cost printed circuit board-based centrifugal microfluidic platform for dielectrophoresis

**DOI:** 10.1038/s41378-024-00856-5

**Published:** 2025-01-27

**Authors:** Nicklas Rondot, Songyuan Yan, Dario Mager, Lawrence Kulinsky

**Affiliations:** 1 Institute of Microstructure Technology, Karlsruhe Institute of Technology, Karlsruhe, Germany; 2 Henry Samueli School of Engineering, University of California, Irvine, CA USA

**Keywords:** Electrical and electronic engineering, Microfluidics, Electronic devices

## Abstract

In recent decades, electrokinetic handling of microparticles and biological cells found many applications ranging from biomedical diagnostics to microscale assembly. The integration of electrokinetic handling such as dielectrophoresis (DEP) greatly benefits microfluidic point-of-care systems as many modern assays require cell handling. Compared to traditional pump-driven microfluidics, typically used for DEP applications, centrifugal CD microfluidics provides the ability to consolidate various liquid handling tasks in self-contained discs under the control of a single motor. Therefore, it has significant advantages in terms of cost and reliability. However, to integrate DEP on a spinning disc, a major obstacle is transferring power to the electrodes that generate DEP forces. Existing solutions for power transfer lack portability and availability or introduce excessive complexity for DEP settings. We present a concept that leverages the compatibility of DEP and inductive power transfer to bring DEP onto a rotating disc without much circuitry. Our solution leverages the ongoing advances in the printed circuit board market to make low-cost cartridges (<$1) that can employ DEP, which was validated using yeast cells. The resulting *DEPDisc* platform solves the challenge that existing printed circuit board electrodes are reliant on expensive high-voltage function generators by boosting the voltage using resonant inductive power transfer. This work includes a device costing less than $100 and easily replicable with the information provided in the Supplementary material. Consequently, with *DEPDisc* we present the first DEP-based low-cost platform for cell handling where both the device and the cartridges are truly inexpensive.

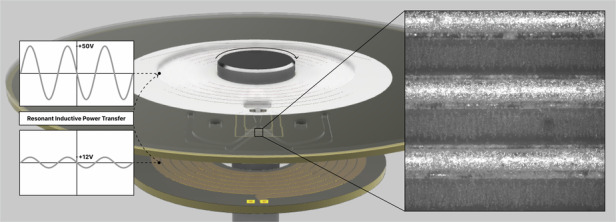

## Introduction

Within the last 20 years, biological testing has increasingly adopted single-cell genomics and proteomics. Next-generation sequencing can provide information about the genome of a single cell^1^. However, sequestering and separation of individual biological cells from the sample presents a challenge and added cost^2^. Dielectrophoresis (DEP) and similar electrokinetic manipulation of biological cells present a low-cost and scalable option for high-throughput biological assays^3,4^. Electrokinetic guidance of particles and biological cells is achieved by utilizing electrodes that generate spatially varying electric fields, inducing dipole moments in suspended cells. Through the dipole moment, the cells experience a force along the field lines, enabling precise control over cell movement and facilitating efficient sorting and manipulation in a microfluidic environment. A quantitative study by Chen et al. revealed that DEP provides efficient single-cell isolation with high yields^5^. The electrokinetic guidance successfully operates with small (microliters) sample volumes, low power requirements (several volts), and low cost, making it an attractive choice for integration within point-of-care bioassay platforms.

Sample preparation steps such as blood separation, dilution, reagents mixing, homogenization, metering, and valving can be significantly simplified when CD microfluidics is used instead of conventional stationary lab-on-chip fluidic assay platforms^6–8^. While CD microfluidic technology can significantly simplify fluidic operations on the platform and integrate sample preparation steps within the same disposable microfluidic disc, in order to use DEP for biological cell handling, electrical power must be delivered onto the spinning disc. Slip-ring assemblies can be used to provide power to a spinning disc^9,10^. One prominent example of the use of a slip-ring assembly is the SpinDEP platform by Martinez-Duarte et al. which leveraged the combined strengths of DEP and centrifugal microfluidics to filter bioparticles^11^. However, the slip-ring brushes degrade over time, the signal is noisy, and the portability and reliability of the test platforms are compromised. The electrified Lab on a Disc (eLoaD) by Torres et al. provided a promising solution in solving the power transfer challenge while maintaining the portability of a centrifugal microfluidic system by implementing inductive power transfer (IPT) through two coils^12–15^. Besides providing a method of transferring power, the eLoaD also integrated a microcontroller underneath the spinning disc that enabled various sensing applications during rotation. While well-suited for various uses, the added complexity and cost of the eLoaD system that is not needed in this application may reduce its attractiveness.

Traditional DEP electrode fabrication involves expensive cleanroom lithography process where a photoresist layer is patterned using a UV source eliminating the photoresist layer through shadow masks^16,17^. Moreover, the method is not widely available for commercial outsourcing at low cost, meaning researchers must fabricate electrodes in-house, which can be costly—many hundreds of US dollars for a set of prototype electrode arrays on a single 4-inch wafer.

In recent years the cost of printed circuit boards (PCBs) has decreased dramatically, making the cost of a custom-ordered PCB with electrical traces comparable or cheaper than the cost of acrylic plastic sheets used for milling of the microfluidic discs. Previously, Mouschou and Tserepi explored the benefits of how PCBs could tackle the upscaling bottleneck of microfluidics and described how various types of sensing electrodes can be integrated using the technology^18^. Perdigones elaborated the flow propulsion mechanisms that can be employed using PCBs^19^. Other groups explored implementing DEP with electrodes fabricated from PCBs^20^. While the PCB was initially regarded as a reusable component and separated from the sample by a surface^21^, ongoing advances in the PCB market have propelled prices low enough that they can be disposed of after use and hence be in direct contact with the sample^22–24^. Consequently, the requirements regarding the voltage are lower, facilitating DEP even with inexpensive function generators.

Today, PCB prototypes can cost less than $5, enabling researchers to test various designs at low cost. Perdigones and Quero presented that the layers of the PCB can be used to fulfill various biomedical functions. The capability to try out various designs at low cost is especially valuable when combined with microfluidics, allowing for innovative cell-handling techniques. Here, we demonstrate how different electrode designs can be manufactured using PCBs. One notable drawback of PCB-based electrodes is the lack of a transparent substrate for less costly fabrication options, making it difficult to observe cells under a microscope. In this work, we will integrate PCB-based DEP electrodes into a centrifugal microfluidic disc and present a fabrication technique that facilitates microscopic observation.

One of the limitations of using PCBs for DEP is the requirement for high-voltage function generators to enable dielectrophoretic forces that are sufficient to move the particles and cells. While electrodes created through lithographic process typically feature gaps of only a few micrometers^25^, the minimum feature size for PCBs is around 100 µm for most manufacturers. As a result, larger electrode gaps on PCBs generate weaker electric fields, leading to reduced DEP forces. To compensate, voltages exceeding 50 V are necessary, which often require expensive function generators or power amplifiers, costing over $5000^21,22^. Consequently, although using PCBs for integrated DEP platforms reduces the cost of the consumables, the requirement for expensive function generators increases the cost of the platform. To address this challenge, we propose the use of resonant IPT, which boosts the voltage of a standard function generator thus avoiding the requirement for use of more expensive equipment.

We introduce an open-source platform that leverages direct IPT to the DEP electrodes, allowing to use a single microfluidic PCB spinning disc to receive electrical power enabling on-board electrokinetic manipulation of particles and biological cells. This work builds upon the previous explorations of wireless DEP^26–28^.

## Results

### A low-cost fabrication technique for DEP electrodes

**Fig. 1:**
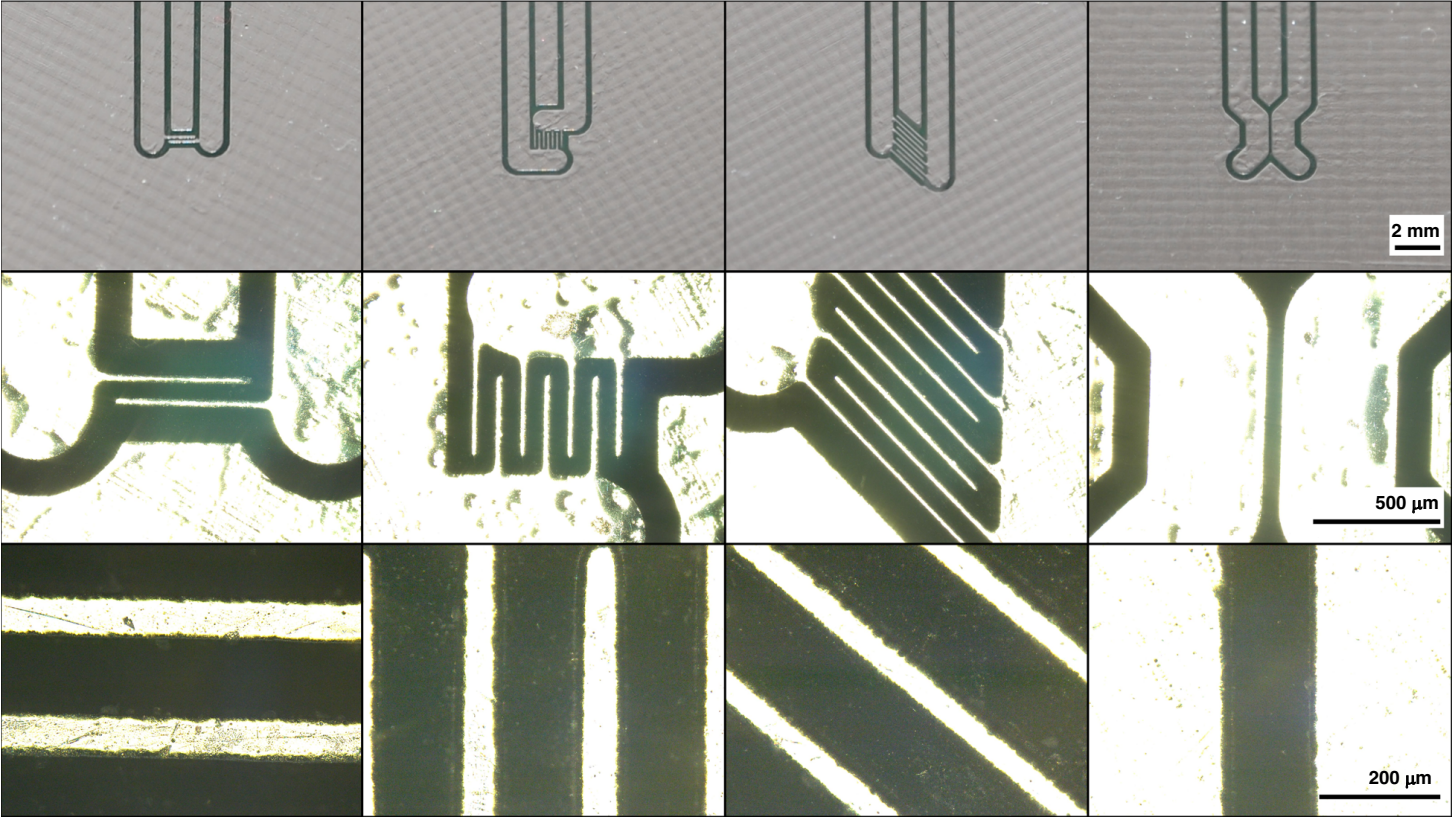
**Various electrode configurations were manufactured from a PCB.** The electrodes are covered in a HASL surface finish, making them reflective to allow observation of cells

We fabricated various electrode configurations on PCBs, as shown in Fig. 1. The minimum trace widths and spacings, which determine the electrode dimensions and gaps, depend on the specific PCB manufacturer. PCB designs were fabricated by JLCPCB (JiaLIChing (Hong Kong) Co. Ltd., Shenzhen, China) with a minimum electrode width and spacing of 100 µm. Microscope observations revealed that the actual trace width ranged between 70 and 80 µm, while the gap measured between 120 and 130 µm.

**Fig. 2:**
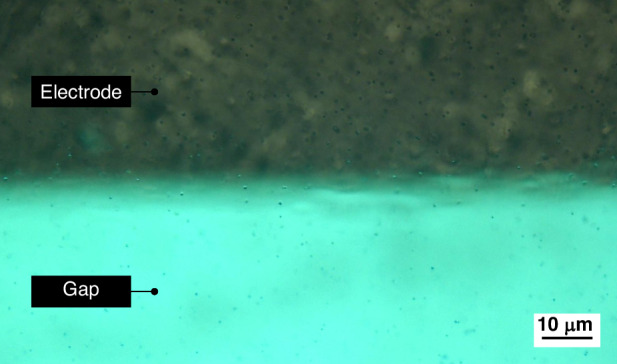
Placement of a light source below the PCB to shine through the fiberglass allows spotting cells in the gaps between the electrodes

Typically, cells situated between the electrodes are difficult to spot due to the high reflectivity of the electrodes compared to the fiberglass substrate (FR4). This issue can be mitigated by avoiding the application of soldermask above the glass fiber areas intended for observation. This allows placing a light source beneath the PCB, shining through the fiberglass to illuminate the cells, as depicted in Fig. 2.

To facilitate the observation of cells on specific regions of the PCB besides the electrodes, we recommend adding a copper plane (not connected to the electrodes) in those areas. By selecting a HASL (silvery solder paste) surface finish and omitting the soldermask, a reflective surface is created. Consequently, the visibility of cells under a microscope is improved when a top lighting source is utilized.

### Inductive power transfer to electrodes

The dielectrophoretic force (*F*_*DEP*_) acting on a particle can be described by the equation^29–31^:

$${F}_{{DEP}}=2\pi {r}^{3}{\varepsilon}_{m}{Re}\left[{K}\left({f}\right)\right]\nabla {\left|{E}\right|}^{2}$$

where *r* is the radius of the particle, *ε*_*m*_ is the permittivity of the medium, *K*(*f*) is the frequency-dependent Clausius–Mossotti factor, *E* is the electric field strength, and ∇|*E*|^2^ is the gradient of the square of the electric field magnitude.

The Clausius–Mossotti factor *K*(*f*) is a function of frequency and can be expressed as:

$$K\left(f\right)=\frac{{\varepsilon }_{p}^{* }\left(f\right)-{\varepsilon }_{m}^{* }\left(f\right)}{{\varepsilon }_{p}^{* }\left(f\right)+2{\varepsilon }_{m}^{* }\left(f\right)}$$

where ${\varepsilon }_{p}^{* }(f)$ is the complex permittivity of the particle, ${\varepsilon }_{m}^{* }(f)$ is the complex permittivity of the medium, and the complex permittivity *ε*^∗^(*f*) is given by:

$${\varepsilon }^{* }\left(f\right)=\varepsilon -\frac{j\sigma }{2\pi f}$$

where *j* is the imaginary unit, *ε* is the real permittivity, *σ* is the conductivity, and *f* is the frequency of the applied electric field.

IPT operates based on Faraday’s Law of Induction, which states that a time-varying magnetic field induces an alternating voltage and therefore current in a nearby conductor. The time-varying magnetic field is generated by applying an alternating current (AC) source to a primary coil. The induced voltage (*V*) in a secondary coil can be expressed as^32^:

$$V=-N\frac{d\Phi }{{dt}}$$

where *N* is the number of turns in the secondary coil and Φ is the magnetic flux passing through it.

The efficiency of power transfer between the two coils depends on their mutual inductance *M*, which is given by:

$$M=k\sqrt{{L}_{1}{L}_{2}}$$

where *L*_1_ and *L*_2_ are the inductances of the primary and secondary coils, respectively, and *k* is the coupling coefficient. The coupling coefficient (0 ≤ *k* ≤ 1) quantifies the strength of the magnetic linkage between the coils, with higher values indicating better coupling. Factors such as coil alignment and the distance between the transmitter and receiver affect *k*, and consequently, the overall power transfer efficiency.

The efficiency *η* of an IPT system is governed by the coupling coefficient *k* and the quality factors *Q*_1_ and *Q*_2_ of the transmitter and receiver circuits, respectively. The overall efficiency can be approximated by:

$$\eta =\frac{{k}^{2}{Q}_{1}{Q}_{2}}{1+{k}^{2}{Q}_{1}{Q}_{2}}$$

where the quality factor *Q* of a coil is defined as:

$${Q}=\frac{2\pi{{f}\,{L}}}{R}$$

with *f* as the AC frequency, *L* as the inductance, and *R* as the resistance of the coil. Higher quality factors lead to lower resistive losses, improving the system’s overall efficiency. Generally speaking, higher frequencies in the range of 1 MHz lead to higher quality factors^33^.

The analysis implies that electrodes employing dielectrophoretic forces on a particle or cell can be connected to a secondary coil powered by a primary coil, forming an aircore transformer. By placing the electrodes with the secondary coil on a spinning rotor and the primary coil on a stationary substrate around the rotor shaft, an efficient power transfer to the spinning disc is enacted.

**Fig. 3:**
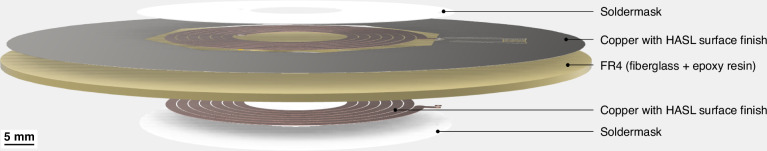
**The receiver disc with the electrodes can be made with traditional PCB fabrication techniques.** The top copper layer integrates both the coil and the electrodes. While the coil can be covered with soldermask to avoid oxidation of the copper, we recommend leaving the electrodes uncovered and using a HASL surface finish. A copper plane also with a HASL surface finish can be placed around the electrodes to allow observation of cells in downstream chambers. The bottom copper layer integrates a coil that continues the one from the top layer to increase the inductance

**Fig. 4:**
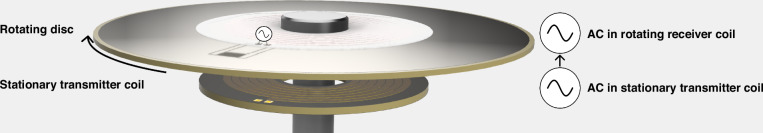
An AC applied to the stationary transmitter coil is inductively transferred to the receiver coil in the rotating disc and conducted directly to the electrodes

A receiver coil is integrated on the microfluidic PCB disc as shown in Fig. 3. This approach is particularly advantageous because it eliminates the need for additional electrical components on the PCB, which would otherwise increase costs and undermine the affordability of PCBs. Given the low cost of PCB fabrication, this integrated design featuring both DEP electrodes and a coil, serves as the foundation for a disposable microfluidic disc. A schematic of the working principle is shown in Fig. 4.

### High-voltage generation through resonance

**Fig. 5:**
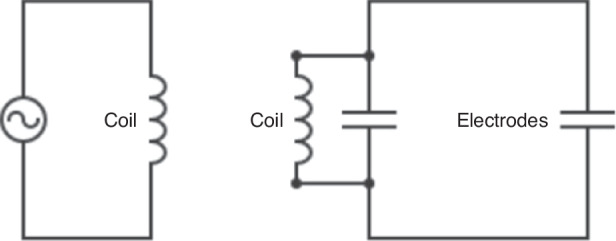
**The transmitter coil couples with the receiver coil.** The electrodes connected to the receiver coil have a low capacitance and consequently allow resonance at high frequencies. To reduce the resonance frequency, the capacitance of the receiver circuit can be altered by adding a second capacitor

To achieve high voltages with a standard function generator to allow employing DEP on a PCB, we introduce the use of resonant IPT. This is implemented using a coil-capacitor network on the receiver (Fig. 5). The resonance frequency *f* is given as:

$$f=\frac{1}{2\pi * \sqrt{{LC}}}$$

where *L* represents the inductance and *C* the capacitance of the circuit. The inherent capacitance of the coil and the DEP electrodes can be leveraged to form such a coil-capacitor network. To alter the capacitance further, an SMD capacitor can be added to the circuit. Alternatively, capacitors can be formed from the copper layers. The overall capacitance *C* can be approximated with the following equations.

$$C={C}_{{electrode}}+{C}_{{coil}}+{C}_{{add}}$$

$${C}_{{electrode}}=n\frac{2\varepsilon {hl}}{{d}_{{electrode}}}$$

$${C}_{{coil}}=\frac{\varepsilon \pi \left({r}_{{outer}}^{2}-{r}_{{inner}}^{2}\right)}{{d}_{{coil}}}$$

**Fig. 6:**
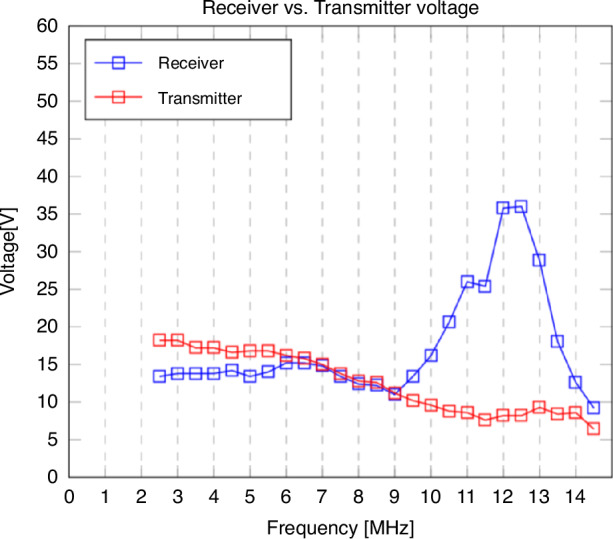
**The voltage in the receiver coil induced by the transmitter coil over the frequency range from 2.5 to 14.5** **MHz.** The transmitter voltage reduces due to limitations of the amplifier. The peak in the receiver voltage implies a resonance frequency of around 12.5 MHz

**Fig. 7:**
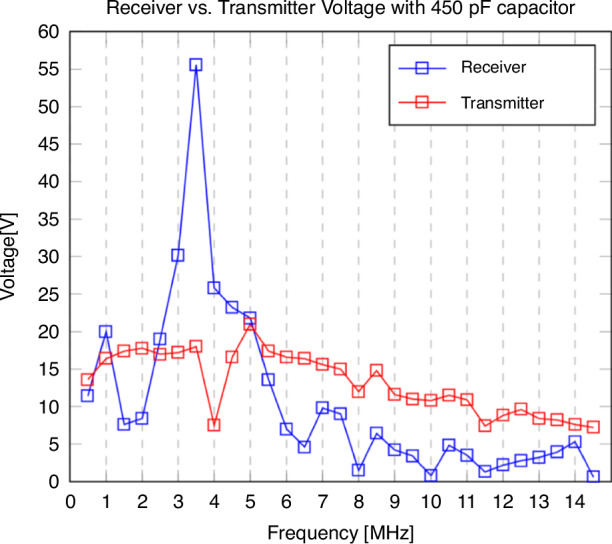
**The voltage in the receiver coil induced by the transmitter coil over the frequency range from 0.5 to 14.5** **MHz after adding a 450** **pF capacitor across the receiver ends.** Due to the increased capacitance, the resonance frequency shifts to 3.5 MHz

Here, *n* is the number of electrode fingers, *ε* the permittivity, *h* the copper layer height, *l* the length of an electrode finger, *d*_*electrode*_ the distance between the fingers, *r*_*outer*_ the outer coil radius, *r*_*inner*_ the inner coil radius, and *d*_*coil*_ the distance between the copper layers. The measured transmitter and receiver peak-to-peak voltage over the frequency is depicted in Fig. 6. Analysis of the curve indicates a capacitance of 35 pF. After placing a 450 pF capacitor across the ends of the receiver coil, the resonance frequency is shifted to 3.5 MHz with a peak-to-peak voltage of 55.6 V (Fig. 7). As the current provided by the amplifier is limited to a few milliamperes, no increase in temperature on the transmitter and receiver coil was observed.

### Dielectrophoresis on a PCB

**Fig. 8:**
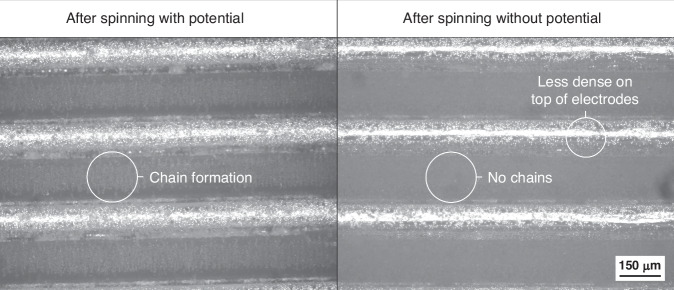
**The disc was spun with 1000** **RPM for 1** **min.** For the left image, a 7 MHz 20 Vpp signal was applied, whereas for the right image no potential was applied during spinning. The images were captured directly after spinning and stopping the potential

To demonstrate the effectiveness of the active dielectrophoretic forces generated by the inductively powered electrodes, experiments using a validation setup with non-resonant IPT were conducted as described below. The validation setup used PCBs with a green soldermask. A 7 MHz, 20 Vpp signal was applied to the transmitter coil using a function generator and then transferred to a receiver disc with interdigitated electrode fingers. Without resonant IPT, this induced a 12 Vpp signal at the electrodes, which is sufficient for the manipulation of polystyrene microbeads. The electrokinetic activity was clearly observed, as the microbeads dispensed on the receiver disc exhibited strong movement toward the electrodes. This motion confirms the presence of active dielectrophoretic forces, as shown in the accompanying video (S1), which illustrates the attraction of microbeads toward the electrodes. Additionally, the ability of the dielectrophoretic forces to counteract centrifugal forces was validated by spinning the receiver disc at 1000 RPM while applying a 7 MHz, 20 Vpp signal. Observations under a microscope after spinning and stopping the applied signal revealed clearly visible chains of microbeads, indicating dielectrophoretic forces (Fig. 8)^34^.

### An integrated platform

**Fig. 9:**
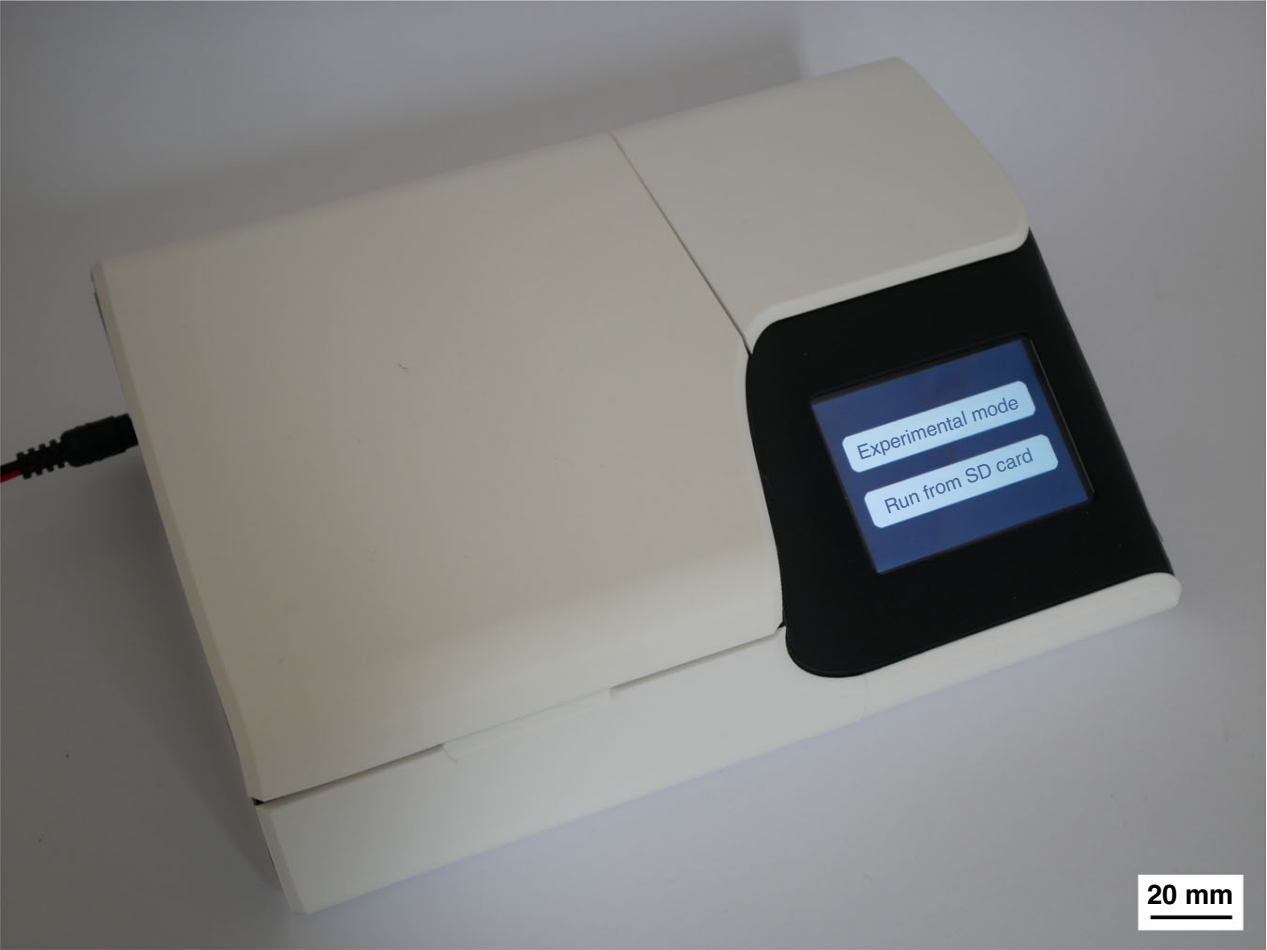
An image of the open-source device providing the capabilities for DEP and centrifugal microfluidics

**Fig. 10:**
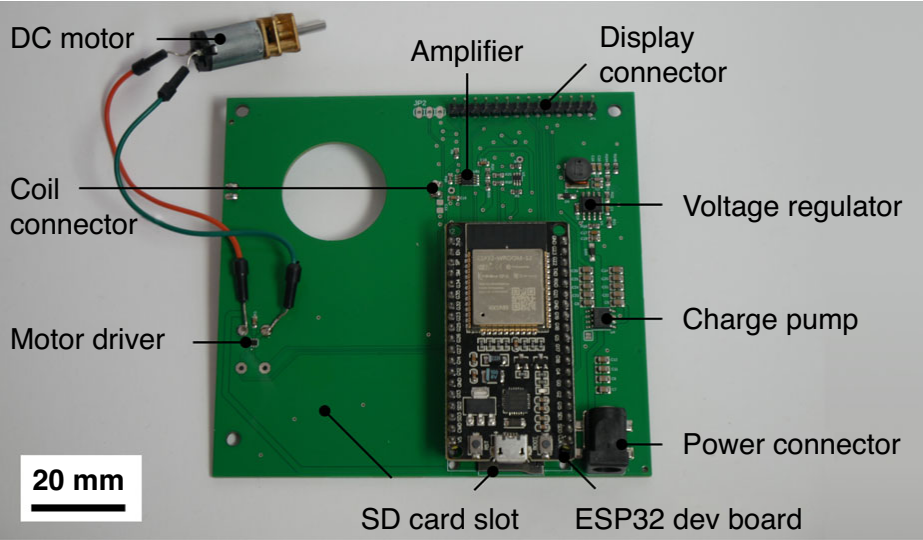
The PCB of the device, integrating a function generator with connectors for a coil and a motor

To make the proposed system where a DEP electrode array on a disposable cartridge is inductively powered usable without the need for a dedicated function generator or a spin stand, an open-source device was developed as presented in Fig. 9. The primary aim of this device is to be easily manufacturable across diverse settings, offering research groups a low-cost, user-friendly solution for experimenting with DEP for cell and particle manipulation and separation. Therefore, the device is designed using only generic, widely available components, and a custom PCB, as depicted in Fig. 10. With a total cost of under $100, the device offers an affordable entry point for DEP research. Comprehensive ordering instructions for the PCB and assembly instructions are included in the Supplementary materials and in the accompanying Github repository: https://github.com/nicklas-rondot/DEPDisc.

The device is controlled via a touch interface and users can set the applied AC frequency and spin speed via USB. Alternatively, users can pre-program a sequence of spin speed/ frequency combinations by creating a text file and loading it through the device’s SD card slot. Various coil designs can be used by connecting a coil PCB with the provided pin headers.

**Fig. 11:**
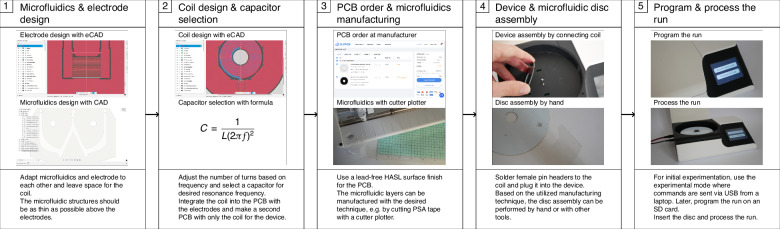
A recommended workflow for designing a DEP application with the DEPDisc platform

The manufacturing technique for electrodes combined with the device forms the *DEPDisc* platform, an integrated platform for DEP-based centrifugal microfluidics. It can be customized by users for various DEP applications via a step-by-step workflow depicted in Fig. 11.

### Cell manipulation

**Fig. 12:**
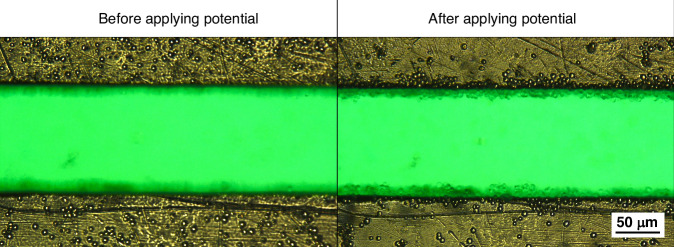
**A 3.5** **MHz, 18** **Vpp AC signal was applied to the transmitter coil, inducing a 3.5** **MHz, 55** **Vpp signal in the receiver disc with the electrodes.** Observation of the gap between two wide electrodes showed that yeast cells are moving towards the edges of the electrodes

To manipulate yeast cells, one typically requires electrodes with small gaps to achieve sufficiently strong electric fields and therefore DEP forces. Here, we demonstrate that our platform allows capturing yeast cells using the electrodes manufactured from a PCB although the larger inter-electrode gaps exceeding 100 µm. As shown in Fig. 12, the yeast cells were attracted to the edges of the electrodes after the AC potential was applied. This proves the platform’s capability to manipulate biological cells, despite fabrication limitations of PCB electrodes.

## Discussion

We envision that the presented platform could serve as a basis for a collaborative community of researchers focused on developing and applying novel DEP technologies. The approach to manufacturing DEP electrodes, as demonstrated here, significantly lowers the barrier to experimentation with diverse designs. By utilizing affordable PCB manufacturers, it is possible to design and receive 10 different electrode designs for just $60. If the PCB is designed to incorporate 12 different electrode configurations (as detailed in the Supplementary information), up to 120 designs can be created for the same cost.

By making this device both open-source and accessible at under $100, we aim to ensure broad replication. This democratization of DEP technology could enable not only well-resourced university labs but also research groups with limited budgets to engage in DEP research and development. This is especially important because these regions benefit the most from a low-cost diagnostic technique hence the local researches have an intrinsic motivation and close insight to the issues they try to solve. As a cost-effective technique, microfluidic PCB-based DEP has the potential to enhance diagnostic solutions in low-resource settings, empowering researchers in those regions to design applications tailored to specific local needs.

Furthermore, this platform provides an opportunity for research groups to explore DEP applications without the need for significant upfront investment in expensive equipment. This is particularly relevant for biochemical labs where DEP is not a core research area but could serve as a valuable tool. With such a low investment needed, one could for example try DEP with a student co-worker. By fostering a community where designs and innovations are shared openly, we aim to streamline DEP adoption, allowing researchers to speed up the trial-and-error phase of electrode design.

While the device we present is based on centrifugal microfluidics, this may not be suitable for every application, particularly those requiring real-time observation during DEP operation, however, its open-source nature encourages modifications. For example, users may choose to adapt it into a more traditional stationary microfluidic system. Additionally, the platform is intended to inspire entirely new systems and reduce development time, for instance by using the custom function generator circuit.

## Conclusion

The presented *DEPDisc* platform was designed and implemented to have both the equipment and the consumables accessible and inexpensive. The key benefit of the platform is that more costly components of the system are placed on a stationary PCB which couples wirelessly to the disposable microfluidic disc with an integrated component-less PCB. Proof-of-concept experiments demonstrated electrokinetic manipulation of yeast cells. By providing detailed instructions for the design, fabrication, and operation of the platform, we aim to allow other research groups to implement their own applications easily. Consequently, the *DEPDisc* platform serves as a low barrier of entry for research groups to get started with DEP experimentation, while the underlying technology is highly scalable.

## Materials and methods

### Microfluidic cartridge disc setup

**Fig. 13:**
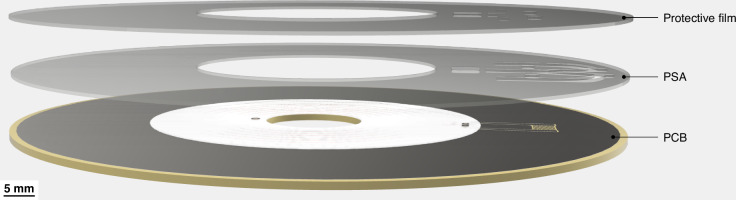
An exemplary layer stack of a microfluidic cartridge with a Disc PCB

The PCB forming the base for the microfluidic disc was manufactured by (JLCPCB JiaLIChing (Hong Kong) Co. Ltd., China). The integrated coil was designed according to the findings presented by Fu et al. ^35^. It has a total of 13 turns with an outer diameter of 22 mm, an inner diameter of 10 mm, a trace width of 1.8 mm, and a turn spacing of 0.2 mm. The inductance, measured with a BM4070 LCR meter, was 4.6 mH (Camway Trading Ltd., Hong Kong). For the PCB, a thickness of 1 mm was chosen, as it is the lowest standard thickness available at low cost, and makes the microfluidic discs lighter. A PSA layer contains microfluidic channels cut from this layer using a Silhouette 3 cutter plotter (Silhouette Studio America Inc., United States). The PSA layer is joined with the PCB and sealed by a thin protective film, as depicted in Fig. 13. In the presented design, a sample is pipetted into the inlet hole, from where it will be pulled into the channel using capillary force.

### Dielectrophoresis validation setup

Aqueous suspensions of carboxyl-modified latex polystyrene beads (5 µm diameter, Thermo Fisher Scientific, Invitrogen, MA, USA) at an initial concentration of 4 wt.% in their original medium were prepared for dielectrophoretic experiments. The bead suspensions were centrifuged and diluted to 0.39 wt.% using deionized (DI) water. This process involved centrifugation (Eppendorf, Germany) at 1500 rpm for 10 min, followed by the removal of the supernatant with a pipette. The beads were then re-suspended in DI water to achieve the desired final concentration of 0.39 wt.% for experimentation.

A centrifugal spin stand was utilized to carry out the experiments. The CDs were spun using a brushless DC motor (Anaheim Automation Inc., Anaheim, CA, USA), controlled by a DC controller (EZSV23, AllMotion, Union City, CA, USA). To capture the movement of liquid and beads on the spinning CDs, a sequence of images was recorded using a digital camera (aca800-510uc, Basler, Inc., Exton, PA, USA) illuminated by a stroboscopic light (DT-311A, Shimpo Instrument, Cedarhurst, NY, USA). Both the camera shutter and the stroboscopic light were synchronized via a reflective IR beam trigger. A 5 mm strip of reflective aluminum tape (Sigma Aldrich, St. Louis, MO, USA) was affixed to the CD’s edge, while an IR sensor (PBT46U, Banner Engineering Corp., Minneapolis, MN, USA) positioned underneath the disc detected the reflective signal, triggering the camera to capture one image per revolution. The images were subsequently analyzed using the ImageJ software.

For the DEP validation experiments, 20 µL of the 0.39 wt.% bead solution was placed onto the PCB disc. A function generator (Siglent SDG 2082X) provided an AC power source to the PCB via the transmitter coil. The receiver PCB containing the integrated receiver coil and DEP electrodes was mounted on the rotating spin chuck. Video recordings were captured using Bandicam software (Bandicam 7.1.1.2158, Bandicam Company, Irvine, CA, USA) while the disc rotated at 1000 RPM for 1 min, and the function generator supplied a 7 MHz, 20 Vpp AC signal to the coil. This setup enabled real-time observation of polystyrene bead behavior at the DEP electrodes powered wirelessly during the rotation of the disc. Observations were also conducted with the stationary disc while maintaining wireless power transfer to the electrodes. These stationary experiments were recorded using a Nikon Eclipse microscope (Nikon, Minato, Japan) equipped with a SPOT RT sCMOS camera (Diagnostic Instruments, Inc., Sterling Heights, MI, USA), and SPOT Basic video software (SPOT Imaging, Sterling Heights, MI, USA) was used for video editing. Additional screen captures were recorded using Camtasia 2018 Recorder (TechSmith Software Company, East Lansing, MI, USA).

### Device setup

The components used in the instrument are listed in Table 1. All electronic components except for the custom PCB are available from a large number of suppliers. The custom PCB was designed in Fusion 360 (Autodesk Inc., San Francisco, CA, USA) and accommodates a function generator, a transmitter coil, a motor driver, and a voltage regulator. Pinouts to plug in a touch display and an ESP32 NodeMCU development board, as well as soldering pads to connect the N20 motor are provided. To power the ESP32 from a 12 V power supply, the custom PCB includes a TPS54331 step-down voltage regulator (Texas Instruments Inc., Dallas, TX, USA).

**Table 1:** Bill of materials for the device

Name	Description	Price
Custom PCB	PCB with function generator, coil, and motor driver	30.00$
ESP32 NodeMCU	ESP32 microcontroller	6.00$
ILI9341 display	2.8 Inch LCD TFT Touch Display	10.00$
N20 12 V 2000 RPM motor	Geared DC motor	5.00$
Female pin headers	Pin headers for mounting ESP32 on custom PCB	3.00$
M1.2 and M3 screws	Pack of M screws	4.00$
Housing	3D printed parts for housing	9.00$
Jumper wires	Wires for connecting custom PCB with display and motor	2.00$

A negative power rail is implemented with an ICL7662 charge pump (Analog Devices Inc., Wilmington, MA, USA).

The integrated function generator is based on the capability of the ESP32 to generate up to 40 MHz signals. An OPA2810 unity-gain operational amplifier (Texas Instruments Inc., Dallas, TX, USA) is used to amplify the signal. This signal is applied via a transmitter coil with the same design and therefore the same inductance as the coil on the microfluidic disc. The PCB was manufactured and assembled externally (JLCPCB JiaLIChing (Hong Kong) Co. Ltd., Shenzhen, China). The housing was printed on a Bambulab A1 Mini 3D printer (Bambulab, Shenzhen, China) from black and white matte PLA filament. All files are included in the Supplementary materials.

### Cell manipulation setup

Commercially available dried baker’s yeast was suspended in DI water. A drop of the solution was pipetted onto the fourth electrode configuration depicted in Fig. 1 and covered with a glass coverslip.

The completed device was operated by sending the values for the disc spin speed and the applied AC signal frequency from a laptop through USB. A 3.5 MHz, 18 Vpp AC signal was applied to the transmitter coil, inducing a 3.5 MHz, 55 Vpp signal at the electrodes.

## Supplementary information

Supplementary materials

## Data Availability

The data supporting this manuscript are available in the following Github repository: https://github.com/nicklas-rondot/DEPDisc.
