# Supplementary material for: A low-cost printed circuit board-based centrifugal microfluidic platform for dielectrophoresis
Source: Microsyst Nanoeng. 2025 Jan 27;11:23. doi: 10.1038/s41378-024-00856-5 (PMC11770146; doi:10.1038/s41378-024-00856-5)
Supplement: Supplementary file 1 — Supplementary materials [file 41378_2024_856_MOESM1_ESM.zip › Supplementary Materials/README_06Oct24.docx]

# DEPDisc

The DEPDisc platform is the first implementation of dielectrophoresis where both the equipment and the consumables are truly inexpensive. It utilizes CD microfluidics to allow the integration of DEP with other microfluidic operations. All aspects of the DEPDisc platform are open-source, including the fabrication technique for the discs (with example designs) and the device. While the paper presents the underlying theory and the results of the DEPDisc platform, this repository is intended to provide the necessary information to reproduce the platform.

This version is from the 06th of October 2024. A more current version might be available at <https://github.com/nicklas-rn/DEPDisc>.
This Word file is a converted version of the README.md file found in the repository. Therefore, hyperlinks to folders might not work here.

## Table of Contents

- [Discs](#Discs)
- [Device](#Device)
- [How to Run](#How%20to%20Run)
- [Additional Figures](#Additional%20Figures)

## Discs

In the folder [Discs](file:////Discs), the design files for the discs can be found. The PCB files can be used in Eagle or Fusion360 Electronics. [Example Disc PCBs](file:////Discs/Example%2520Disc%2520PCBs) are provided as inspiration. The separate [coil PCB](file:////Discs/Coil%2520PCB/) used as transmitter is included as well. For new designs, the [coil PCB library](file:////Discs/Coil%2520PCB%2520Library/) can be used.

## Device

The [Device CAD and PCB folder](file:////Device/) contains all the necessary files to reproduce the DEPDisc device. The device is composed of a PCB, off-the-shelf components, and a set of 3D printed parts.

### PCB

To order the PCB at [JLCPCB](https://jlcpcb.com), the [Manufacturing folder](file:////Device/PCB/Manufacturing/) with a [ZIP file](file:////Device/PCB/Manufacturing/DEPDisc%2520Base%2520rev1%2520v8_2024-09-17.zip) of the Gerber files is included. For assembly by JLCPCB, the [BOM file](file:////Device/PCB/Manufacturing/DEPDisc%2520Base%2520rev1%2520v8_2024-09-17.zip) and [Pick and Place file](file:////Device/PCB/Manufacturing/DEPDisc%2520Base%2520rev1%2520v8_2024-09-17.zip) are also provided. The ordering process is the follwing:

1. Go to [JLCPCB](https://jlcpcb.com)
2. Click on **Add gerber file** and upload the ZIP file
3. Select the desired options and **Enable PCB Assembly** (choose 2 as PCBA quantity instead of 5). Select **Bottom Side Assembly**.
4. After clicking **Next**, upload the **BOM file** and **CPL (Pick and Place) file**, then continue
5. When reviewing the parts, ensure that only C13 and JP3 show ‘No part selected’ and that the rest of the parts are correctly assigned. Then click **Next** and then **Do not Place** upon the prompt showing that the project has unselected parts.
6. Review the part placement and click **Next**, then click **Save to Cart**
7. Review the order and click **Secure Checkout**

The [design files](file:////Device/PCB/Design%2520Files) of the PCB for Eagle or Fusion360 Electronics are included in case this project is used as inspiration or if modifications are desired.

### Housing

The [parts for printing](file:////Device/3D%2520Printed%2520Parts) are provided as STL files. In the slicer, we recommend enabling tree-supports. A [STEP file](file:////Device/DEPDisc%2520Device%2520rev1.step) of the assembly is included.

### Assembly

To assemble the device, use the following procedure:

1. Place M3 nuts in all the provided slots in the 3D printed parts.
2. Solder the pin-headers to the PCB:

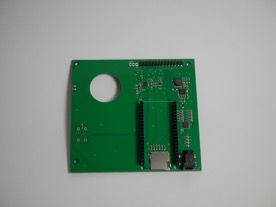

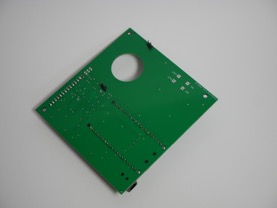

3. Solder two jumper wires to the motor:

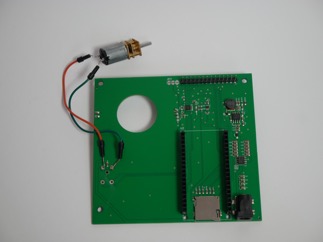

4. Plug the ESP32 NodeMCU Dev board into the pin-headers:

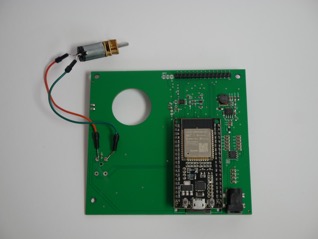

5. Screw the touch display to the display mount:

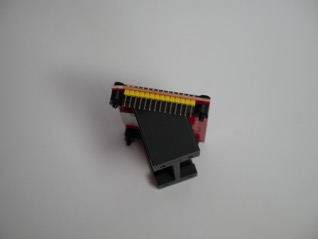

6. Plug in 14 female-to-female jumper wires to the display. We recommend placing tape around the ends to keep the wires together when they are removed:

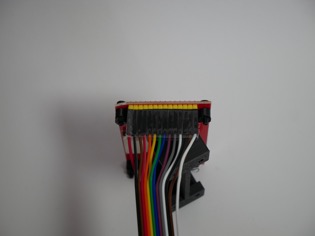

7. Plug the other side of the jumper wires into the PCB.

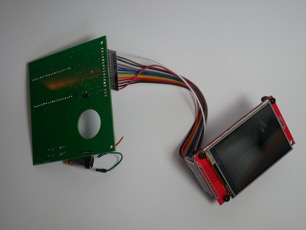

8. Slide the PCB into the left side of the inner shell of the housing and move the motor through the hole:

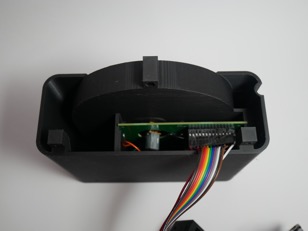

9. Screw the motor in place with two M1.2 screws:

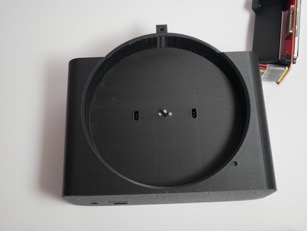

10. Slide the display in to the right side of the inner shell of the housing:

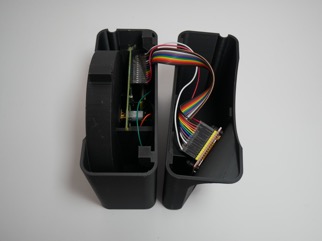

11. Screw the display in place from the bottom with four M3 screws:

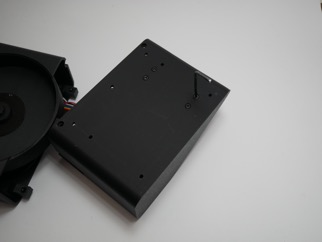

12. Connect the two inner shell sides with three M3 screws:

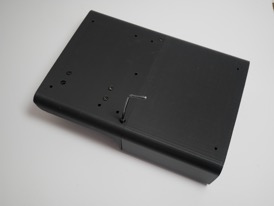

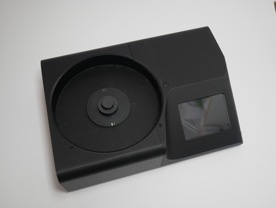

13. Place the right side of the outer shell on top of the inner shell by carefully bending it slightly:

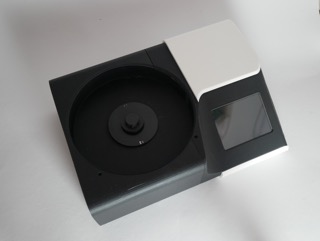

14. Push the aluminium rod through the left side of the outer shell and the hatch:

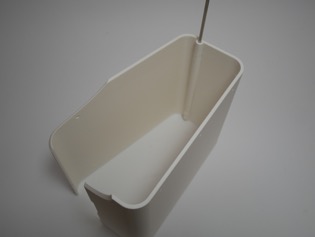

15. Push the left side of the outer shell on top of the inner shell by moving it in from the left and carefully bending it slightly:

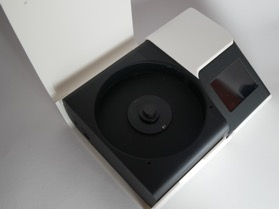

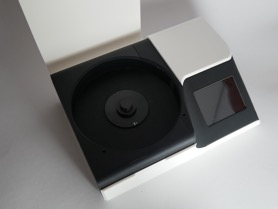

16. Screw six M3 screws into the outer shell:

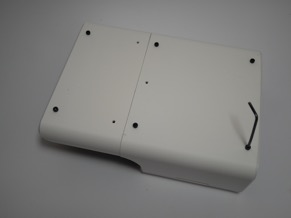


### Programming

To program the device, use the [Arduino file](file:////Device/Software/DEPDisc_base.ino) provided in the [Software folder](file:////Device/Software). Use to following procedure:

1. Download the Arduino IDE
2. Install the ESP32 boards under the boards manager (Second tab from the top, type ‘esp32’ in the search bar, and click install)
3. Click on Tools -> Board -> esp32 -> ESP32 Dev Module
4. Select the correct port under Tools -> Port
5. Click on the upload button (right arrow) to upload the code to the ESP32

## How to Run

### Experimental mode

Connect the device with a USB cable to a laptop. Use the Serial Monitor of the Arduino IDE and select a Baud Rate of 115200. To set a specific frequency, type: freq [frequency in Hz]. To set a specific speed, type: spin [PWM value between -255 and 255]

### Run from SD card

Write a text file with the name **run.txt** to the SD card with the same commands as above. Between the commands use a newline. Use the command delay [delay in seconds] to set delays. Insert the SD card into the device and select ‘Run from SD card’. The device will read the file and execute the commands.

##

## Additional Figures


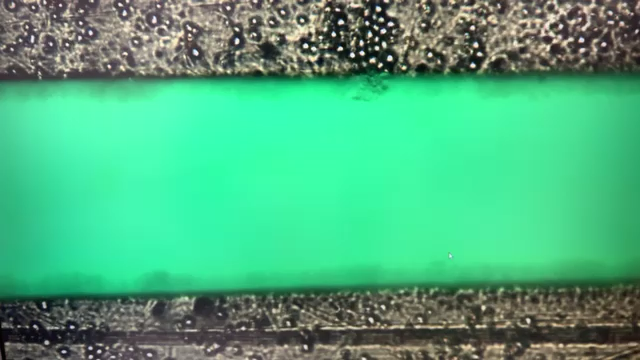


(/Figures/dep_edge.mov)

A video showing the attraction of yeast cells to the edges of two electrodes. A 3.5 MHz signal was applied 6 seconds after the start of the video.
