# Supplementary figures and images for: A low-cost printed circuit board-based centrifugal microfluidic platform for dielectrophoresis
Source: Microsyst Nanoeng. 2025 Jan 27;11:23. doi: 10.1038/s41378-024-00856-5 (PMC11770146; doi:10.1038/s41378-024-00856-5)

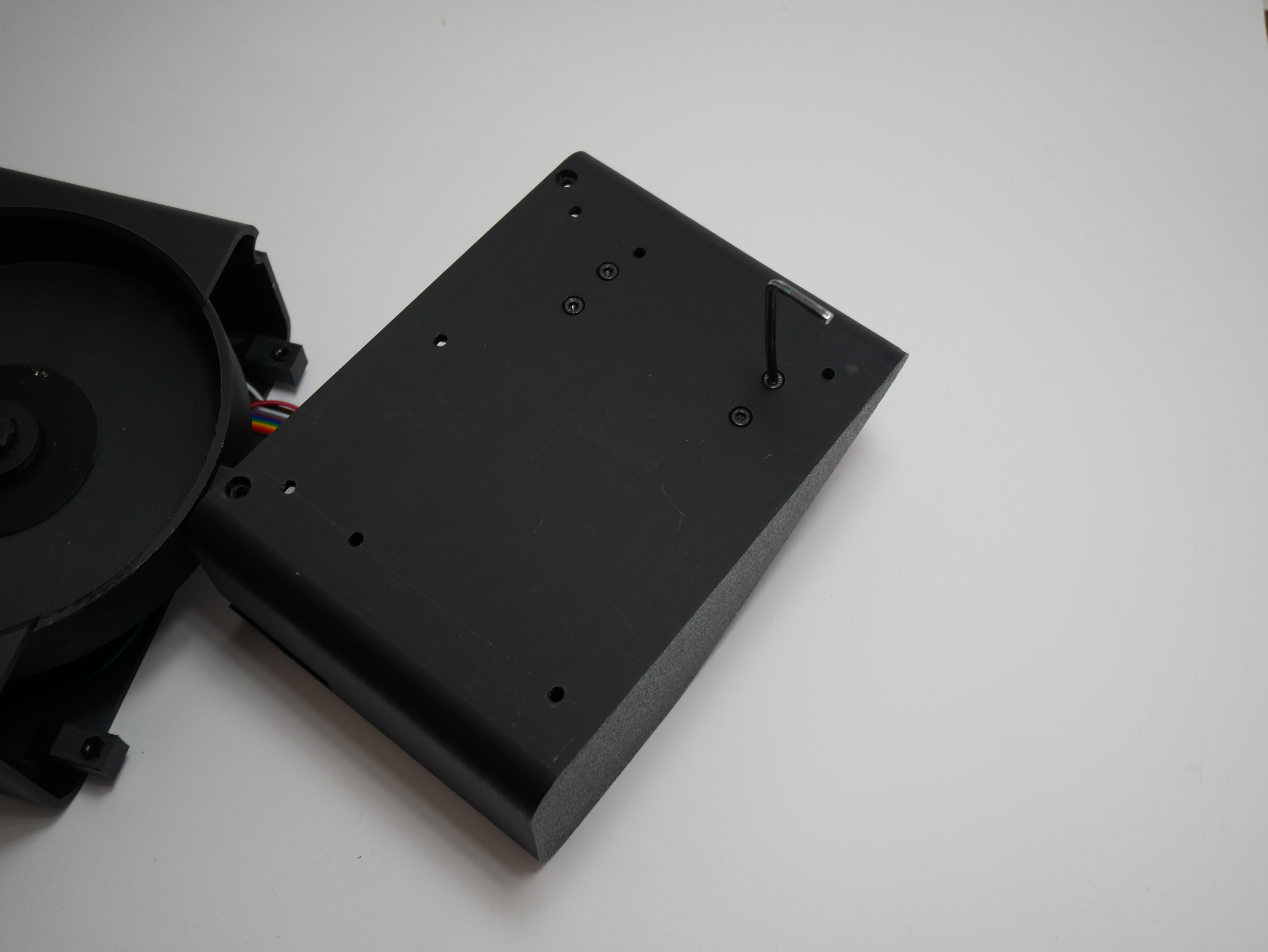

Supplement: Supplementary file 1 — Supplementary materials [file 41378_2024_856_MOESM1_ESM.zip › Supplementary Materials/Figures/display_screws.JPG]

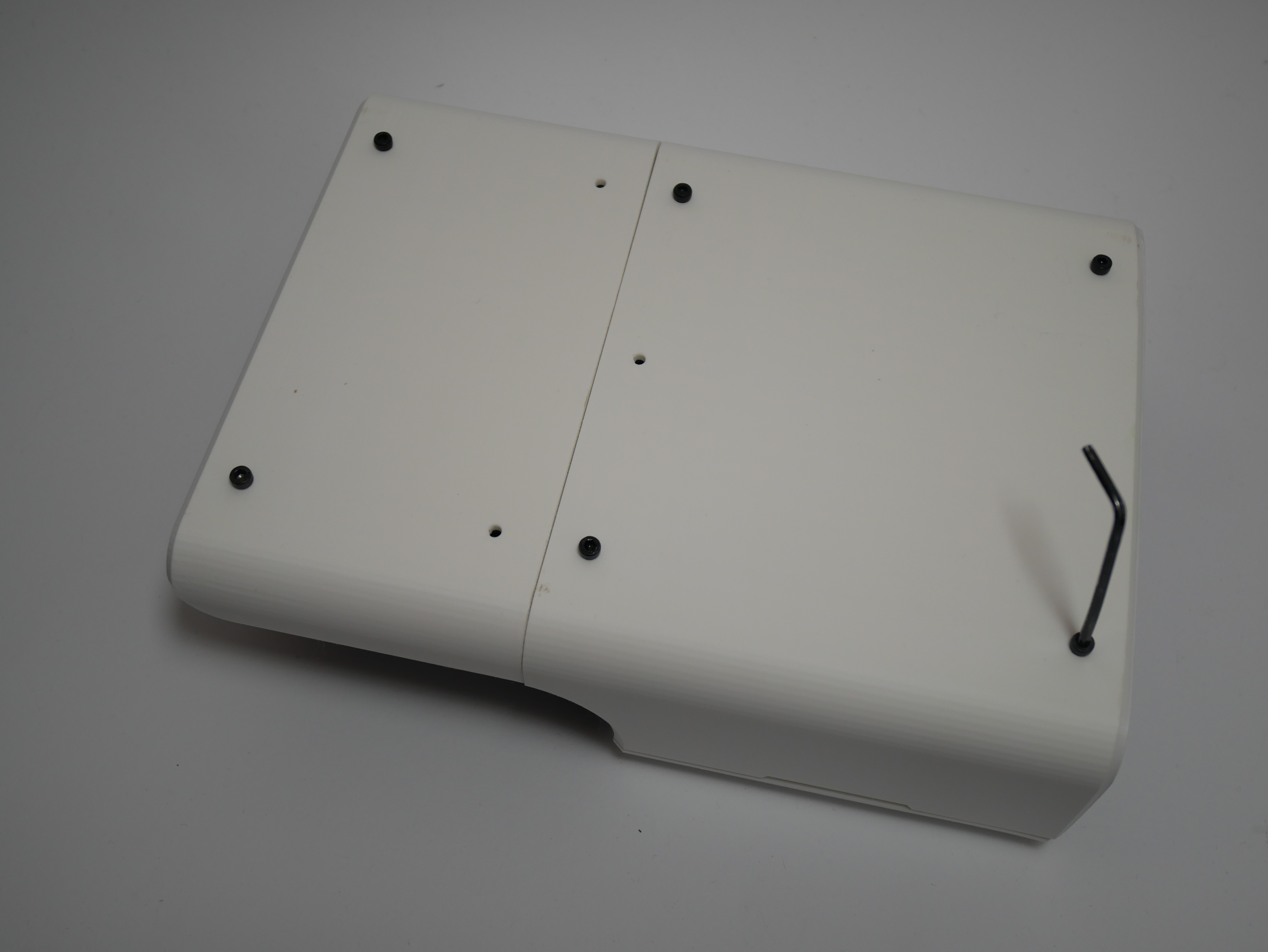

Supplement: Supplementary file 1 — Supplementary materials [file 41378_2024_856_MOESM1_ESM.zip › Supplementary Materials/Figures/shell_screws.JPG]

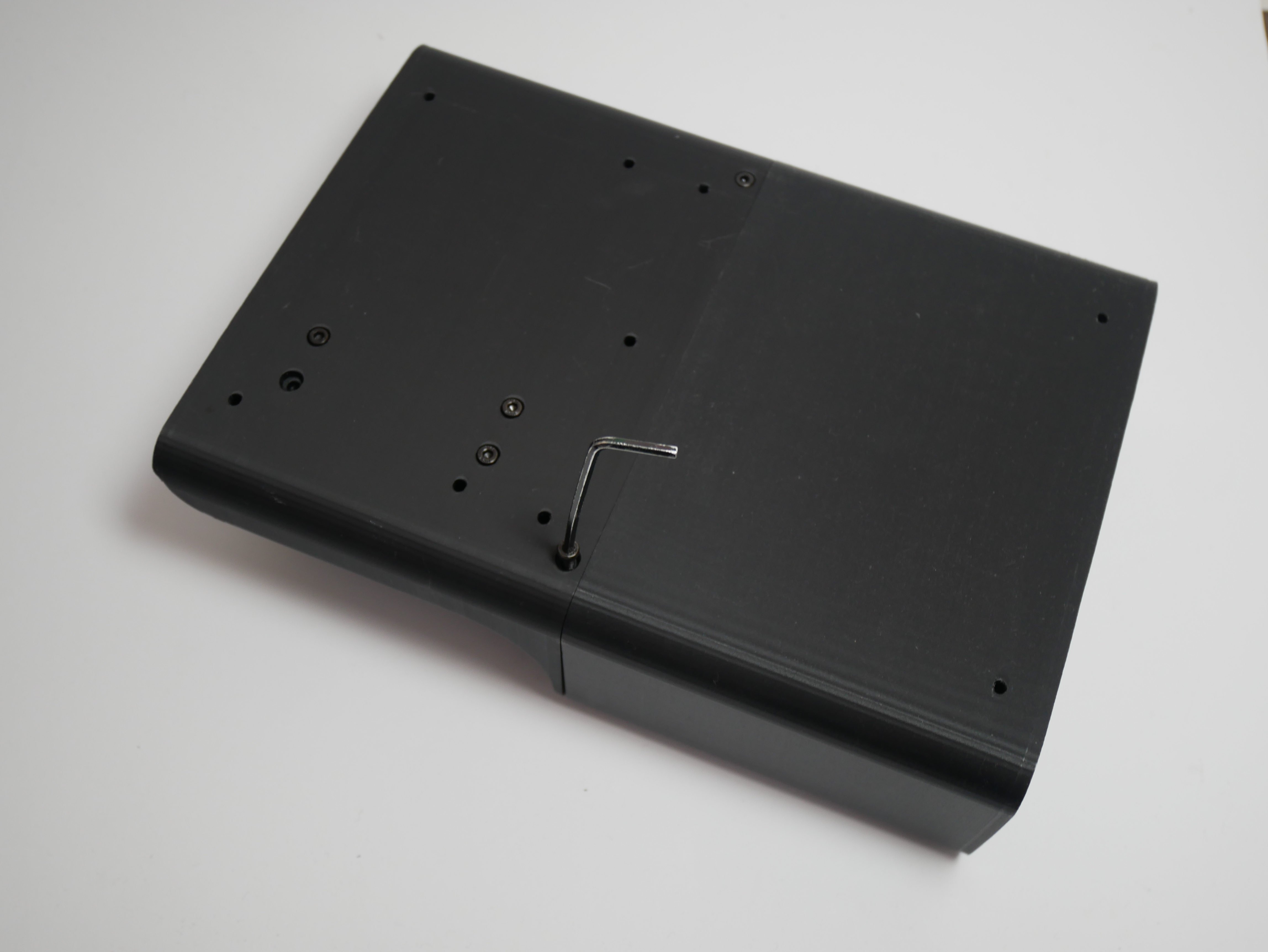

Supplement: Supplementary file 1 — Supplementary materials [file 41378_2024_856_MOESM1_ESM.zip › Supplementary Materials/Figures/housing_screws.JPG]

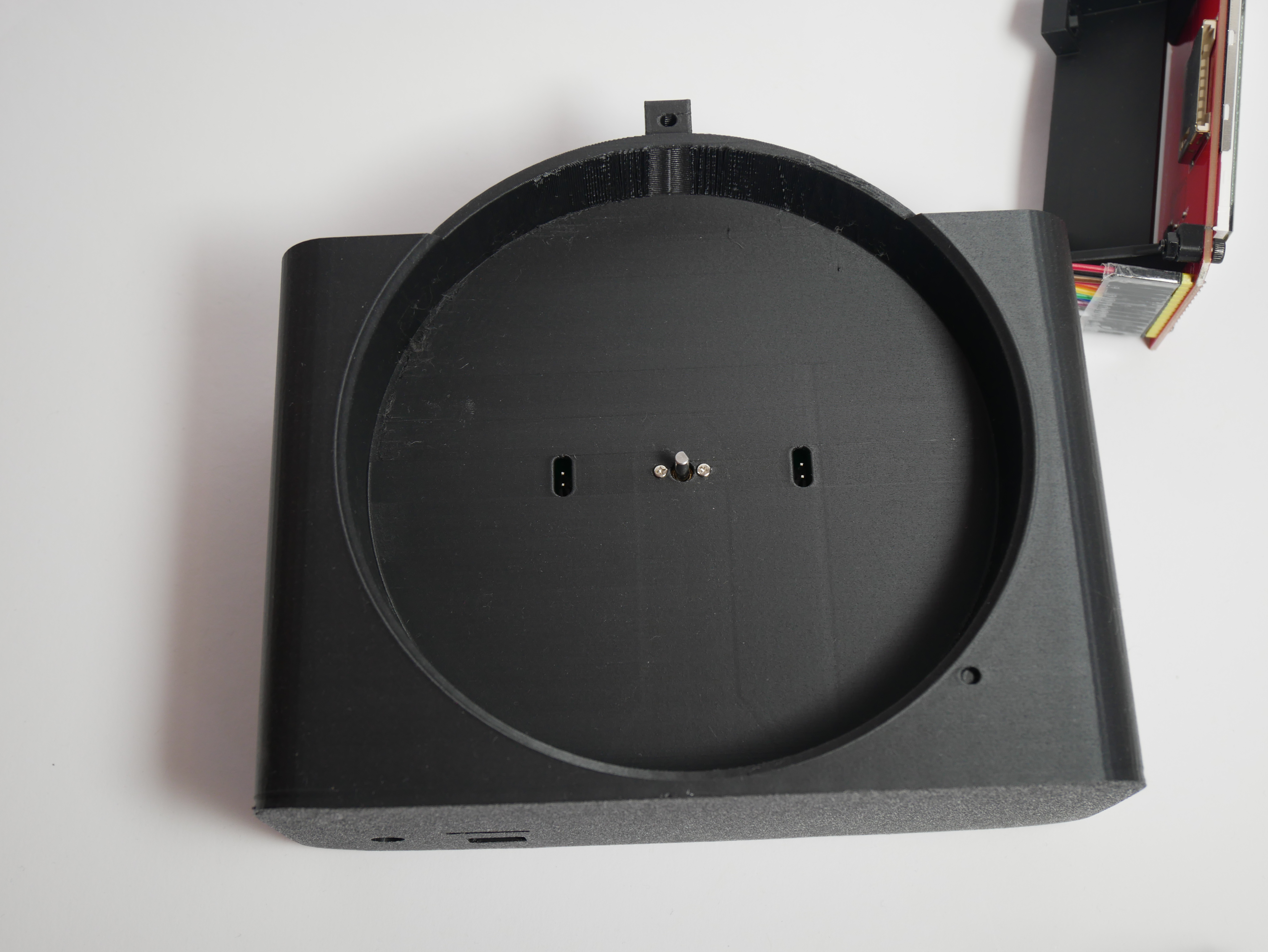

Supplement: Supplementary file 1 — Supplementary materials [file 41378_2024_856_MOESM1_ESM.zip › Supplementary Materials/Figures/motor_screws.JPG]

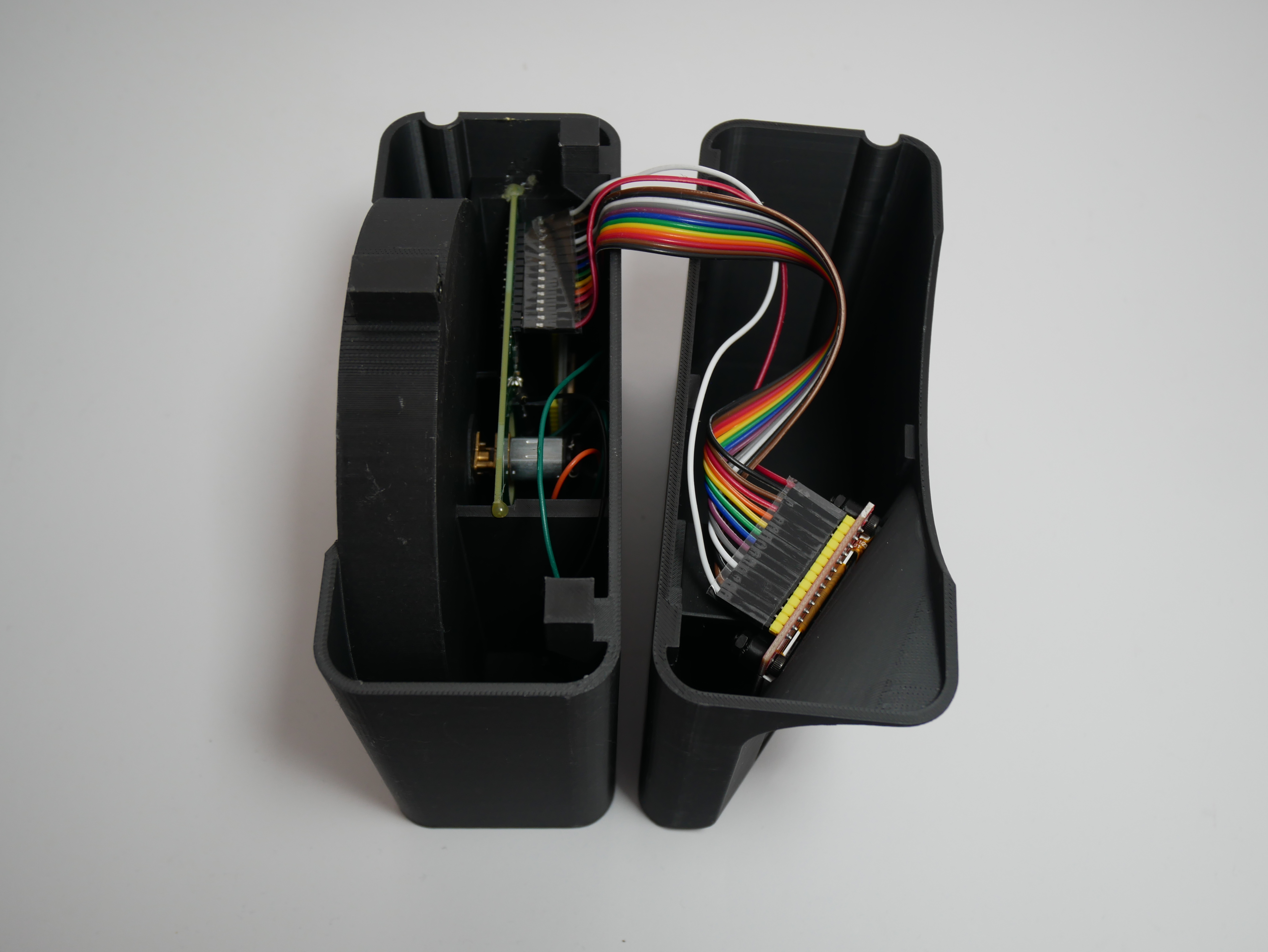

Supplement: Supplementary file 1 — Supplementary materials [file 41378_2024_856_MOESM1_ESM.zip › Supplementary Materials/Figures/display_housing.JPG]

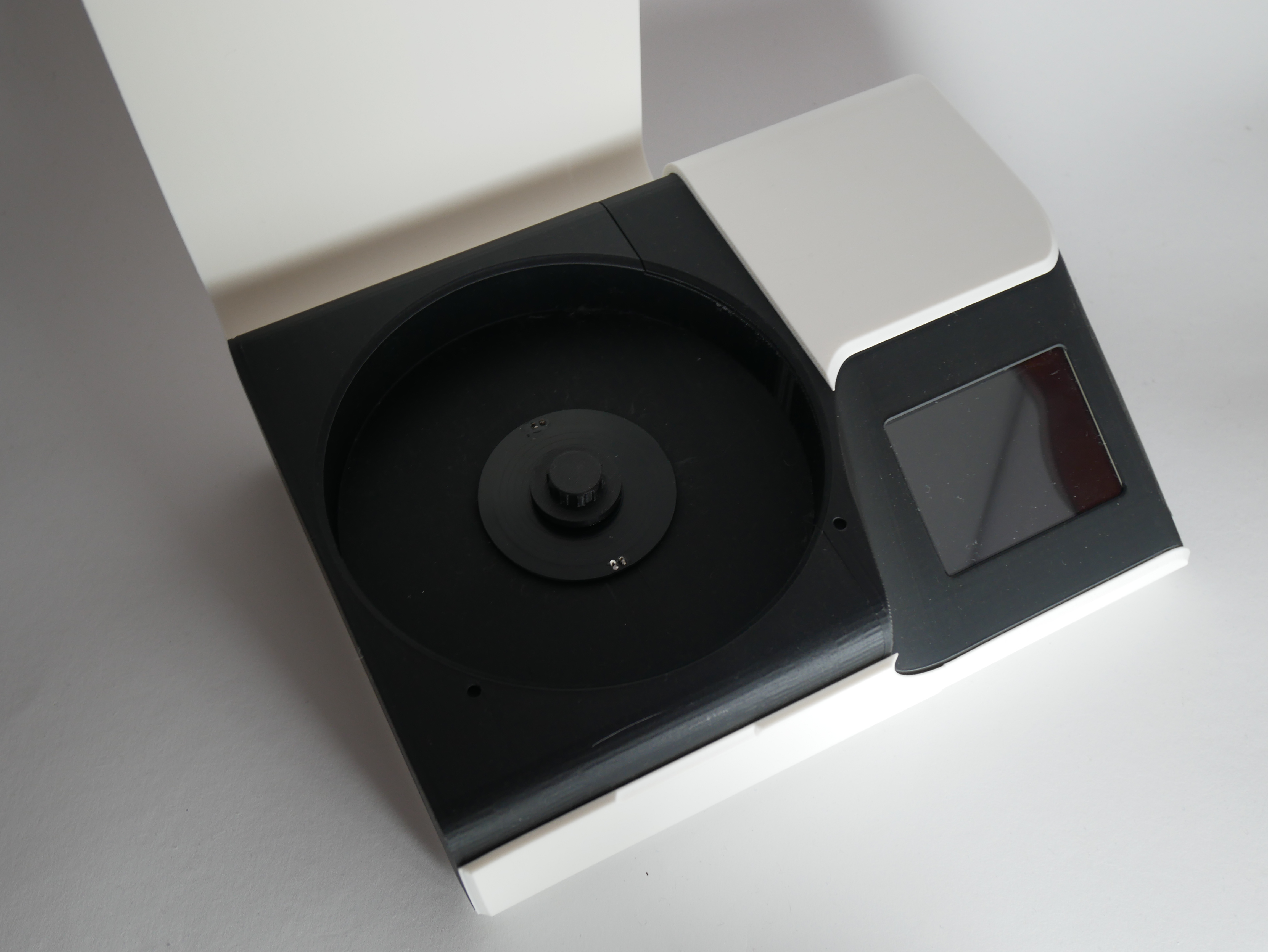

Supplement: Supplementary file 1 — Supplementary materials [file 41378_2024_856_MOESM1_ESM.zip › Supplementary Materials/Figures/left_outer2.JPG]

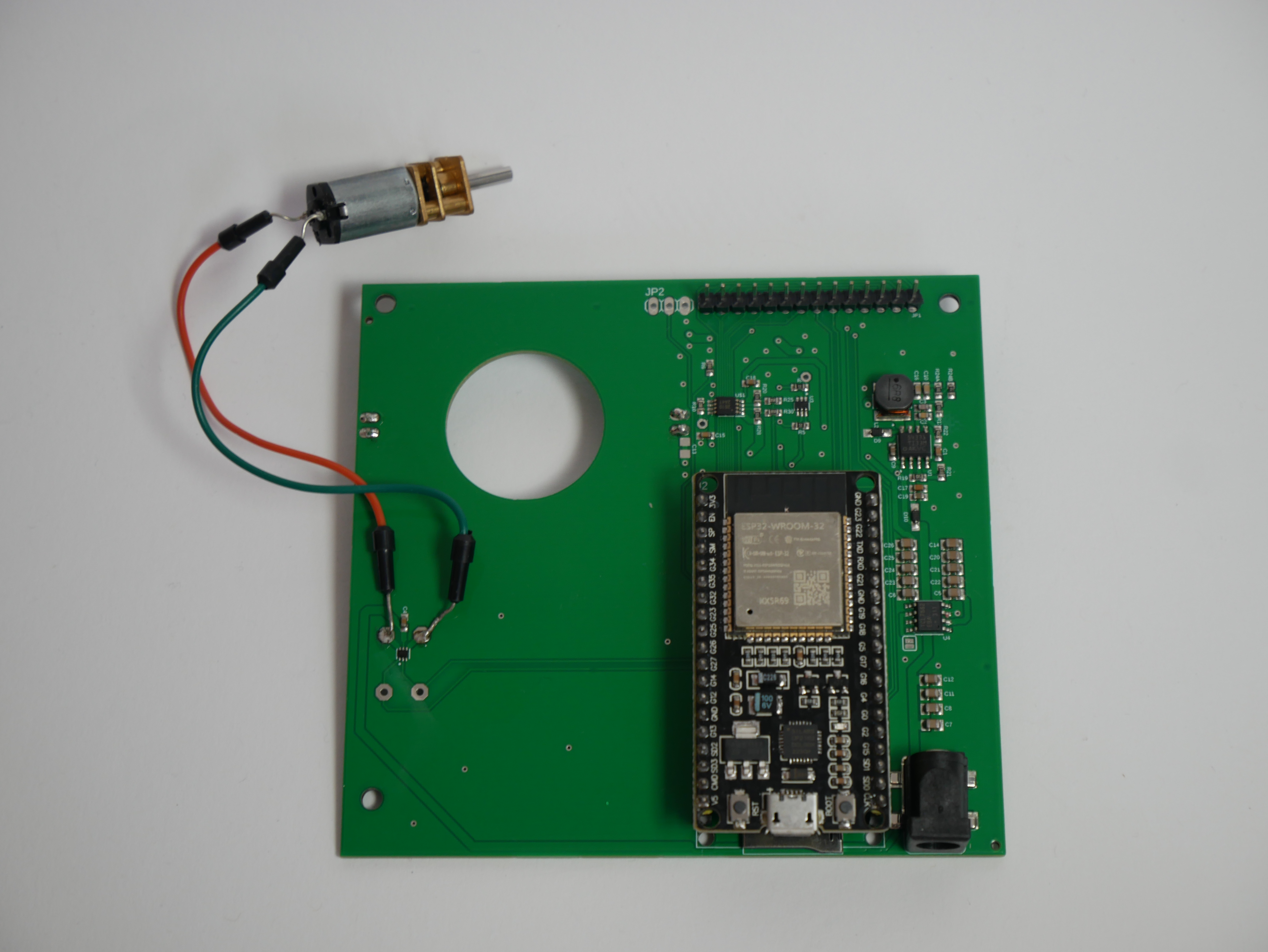

Supplement: Supplementary file 1 — Supplementary materials [file 41378_2024_856_MOESM1_ESM.zip › Supplementary Materials/Figures/pcb_esp32.JPG]

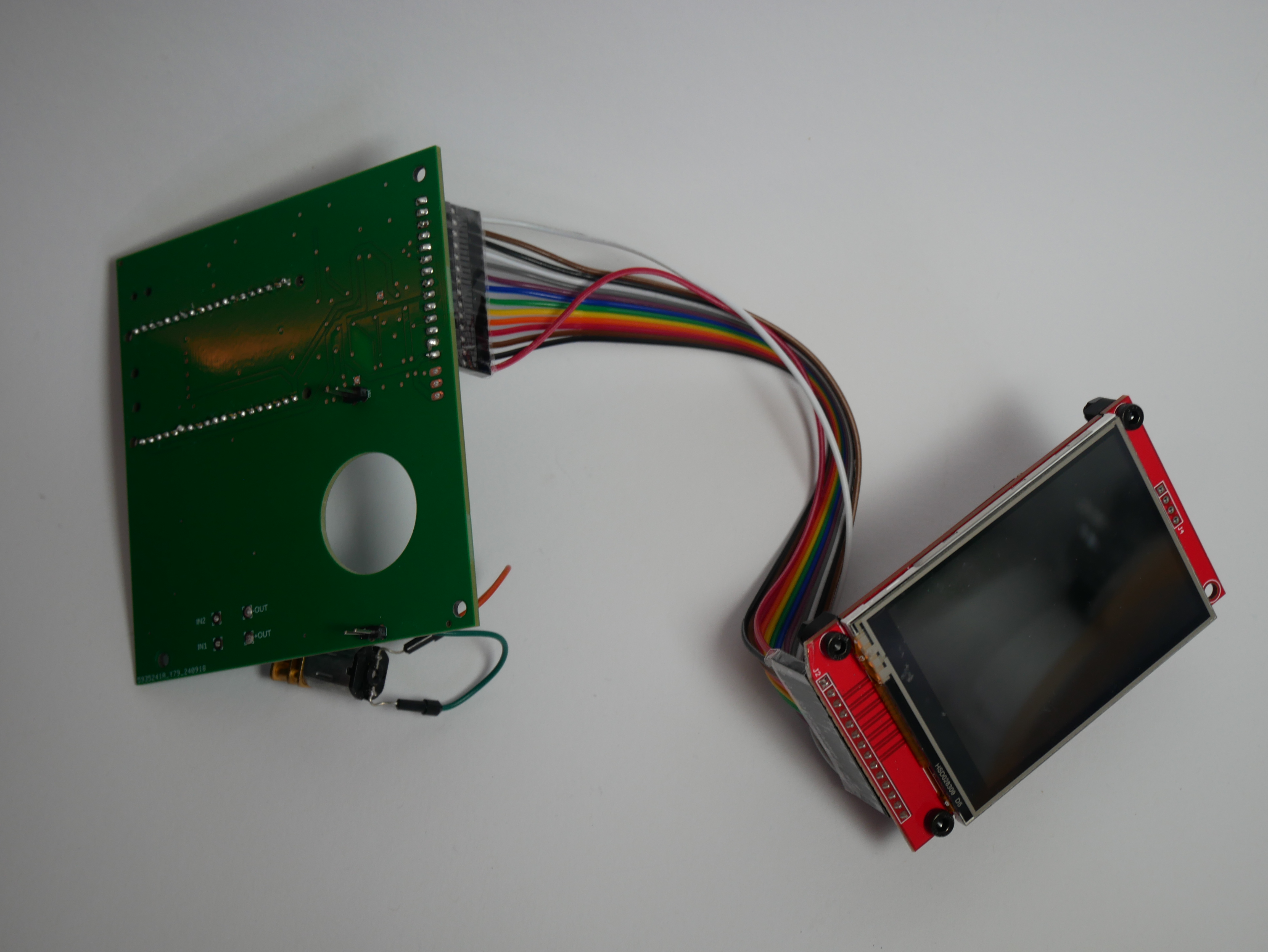

Supplement: Supplementary file 1 — Supplementary materials [file 41378_2024_856_MOESM1_ESM.zip › Supplementary Materials/Figures/pcb_display.JPG]

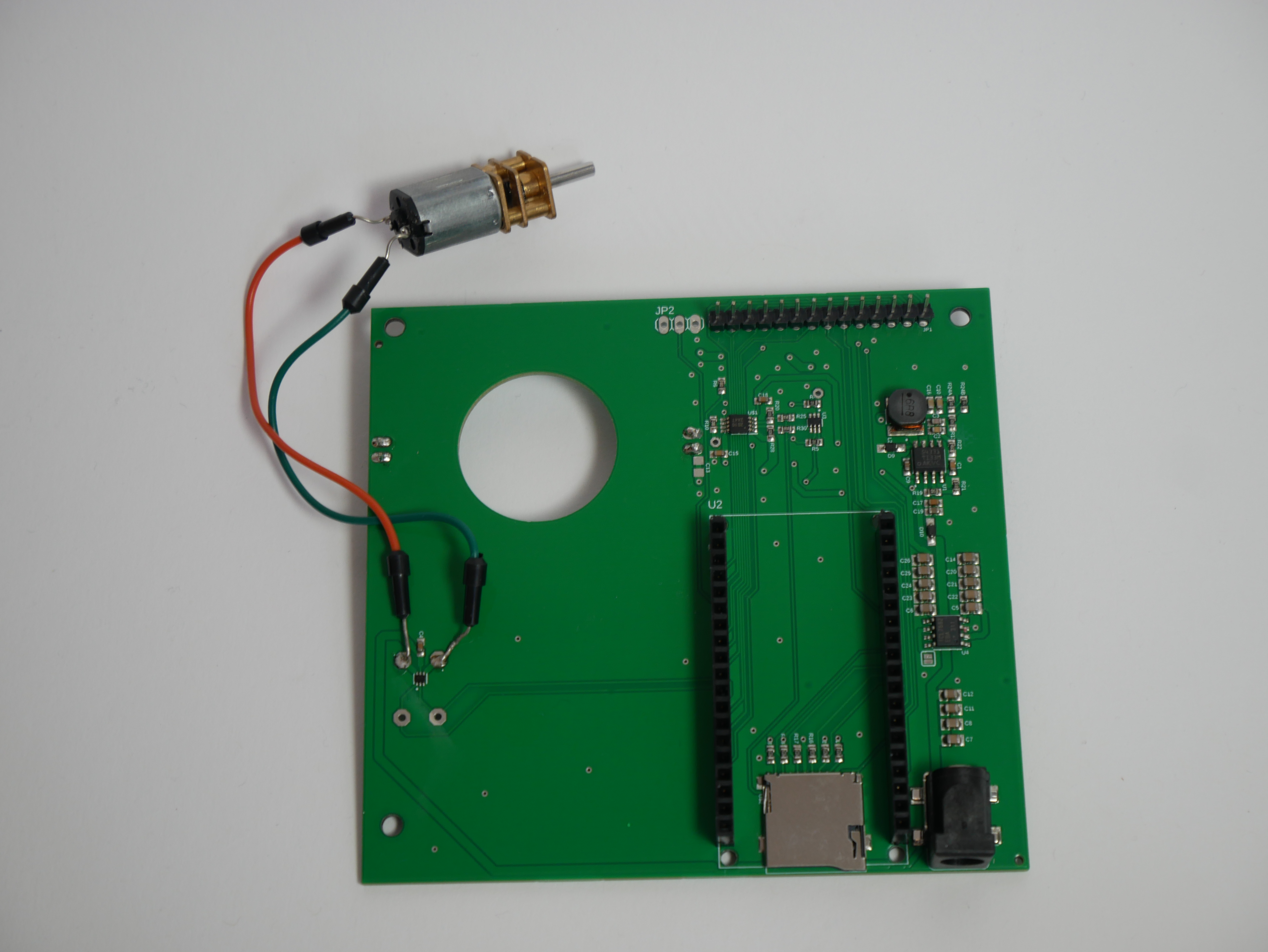

Supplement: Supplementary file 1 — Supplementary materials [file 41378_2024_856_MOESM1_ESM.zip › Supplementary Materials/Figures/pcb_motor.JPG]

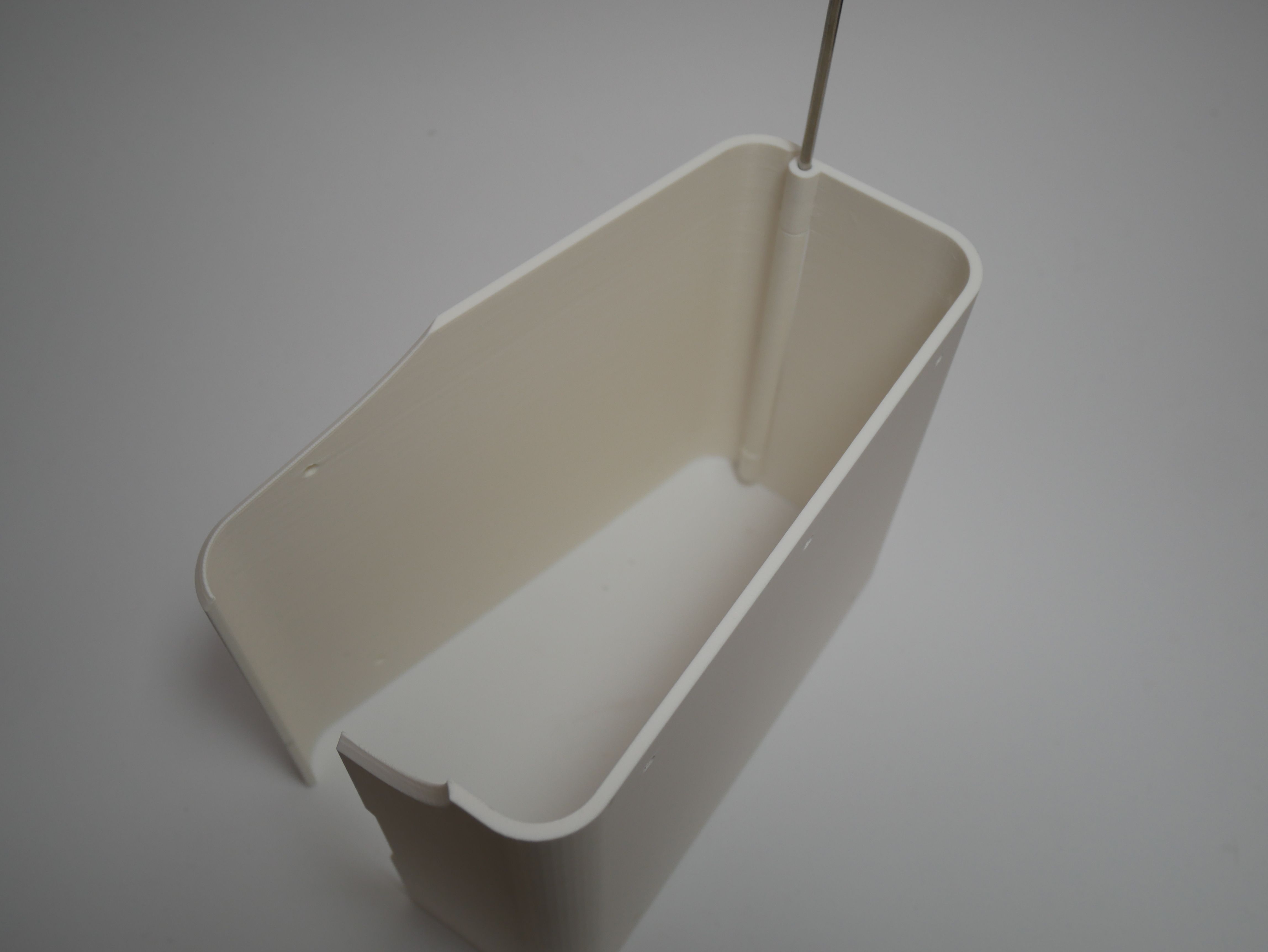

Supplement: Supplementary file 1 — Supplementary materials [file 41378_2024_856_MOESM1_ESM.zip › Supplementary Materials/Figures/rod.JPG]

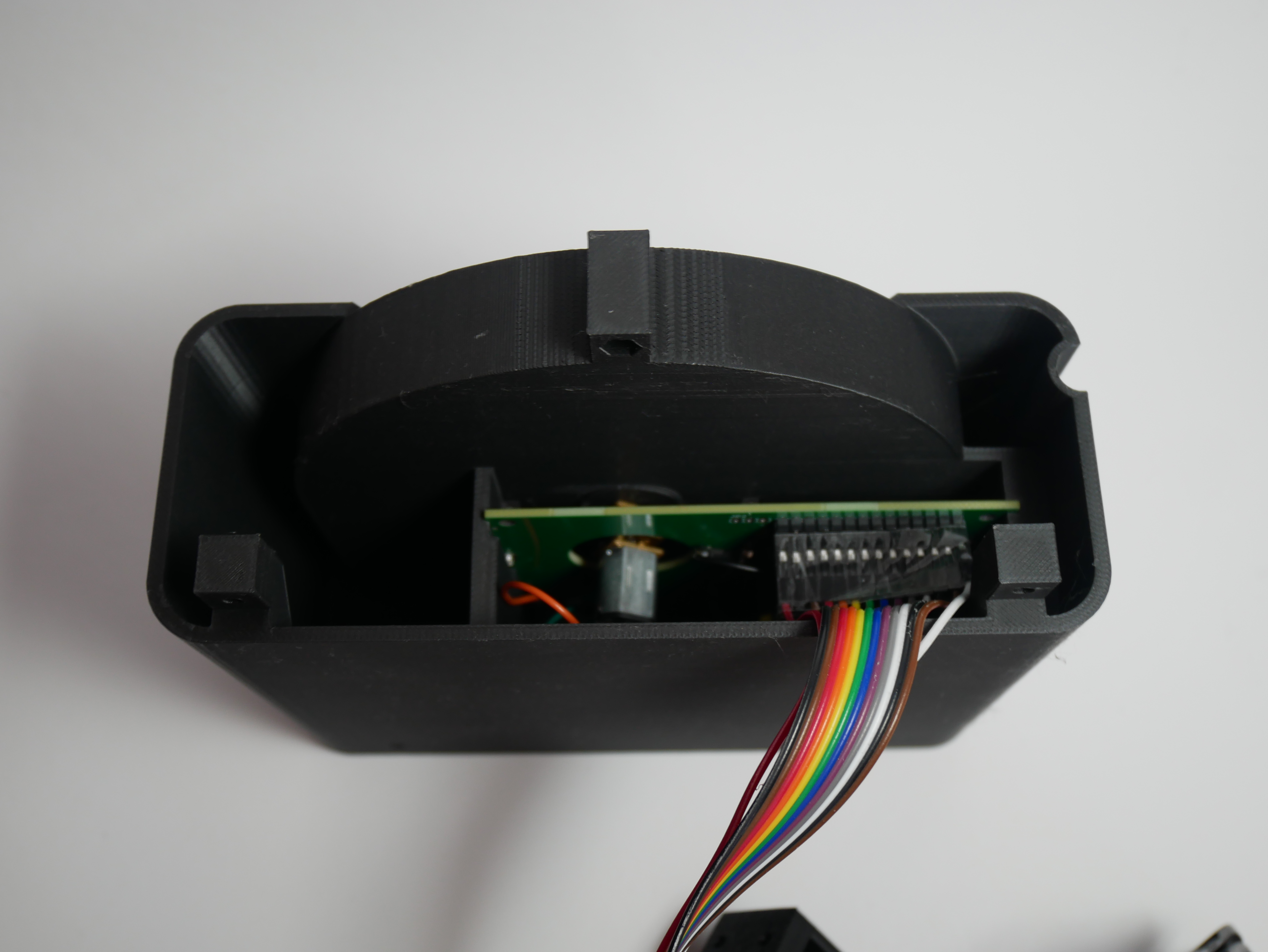

Supplement: Supplementary file 1 — Supplementary materials [file 41378_2024_856_MOESM1_ESM.zip › Supplementary Materials/Figures/pcb_housing.JPG]

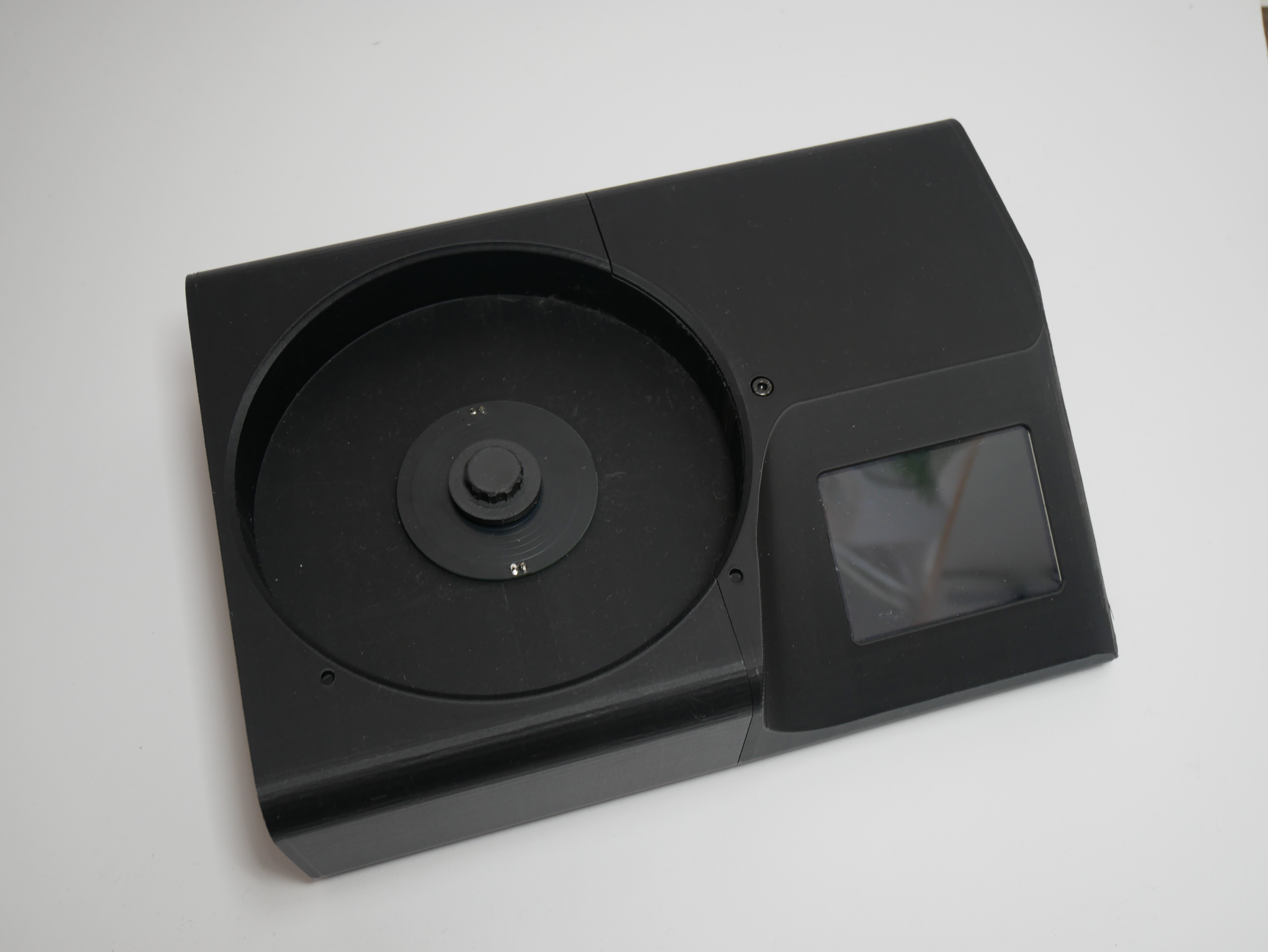

Supplement: Supplementary file 1 — Supplementary materials [file 41378_2024_856_MOESM1_ESM.zip › Supplementary Materials/Figures/housing_screws2.JPG]

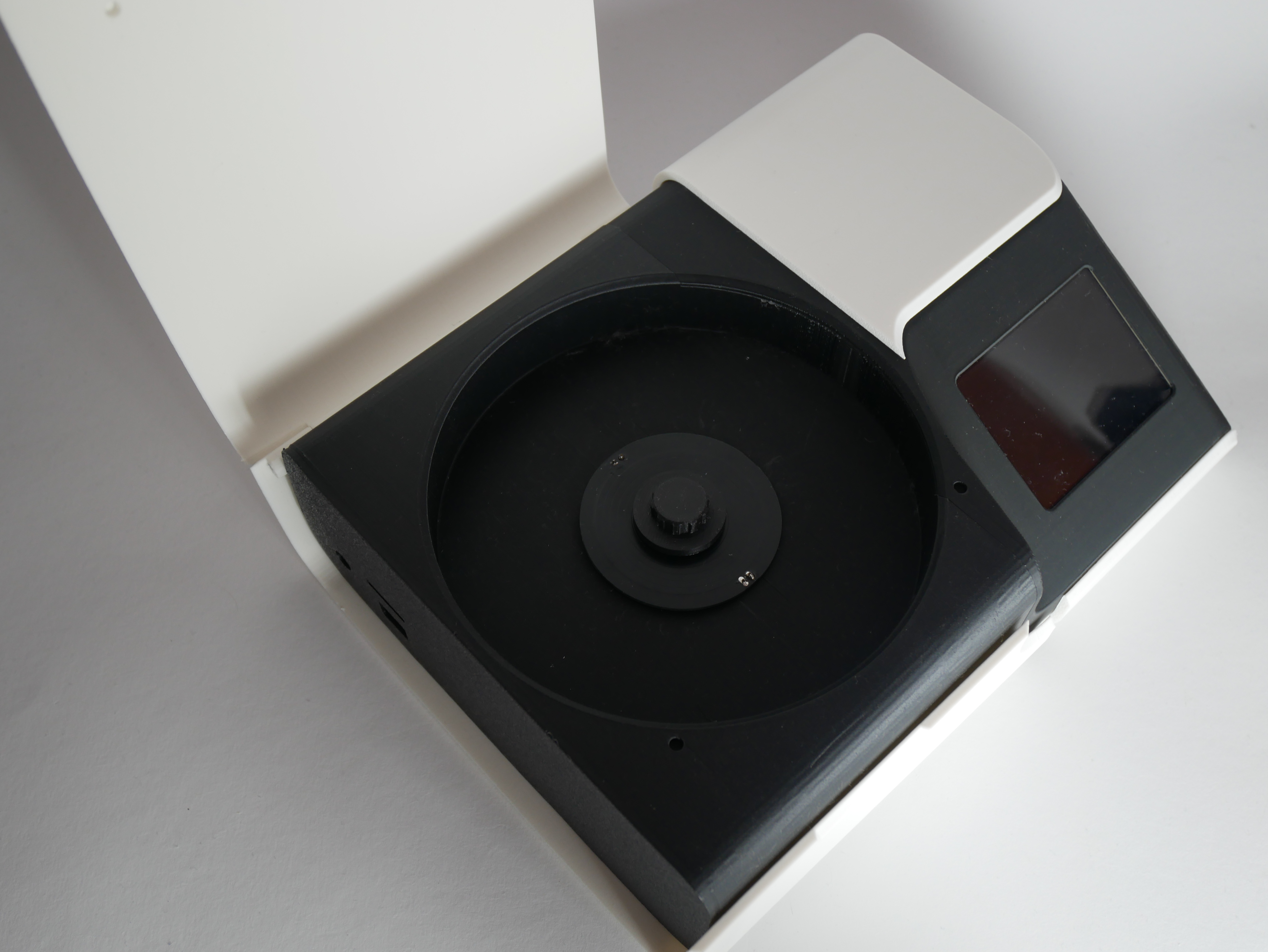

Supplement: Supplementary file 1 — Supplementary materials [file 41378_2024_856_MOESM1_ESM.zip › Supplementary Materials/Figures/left_outer1.JPG]

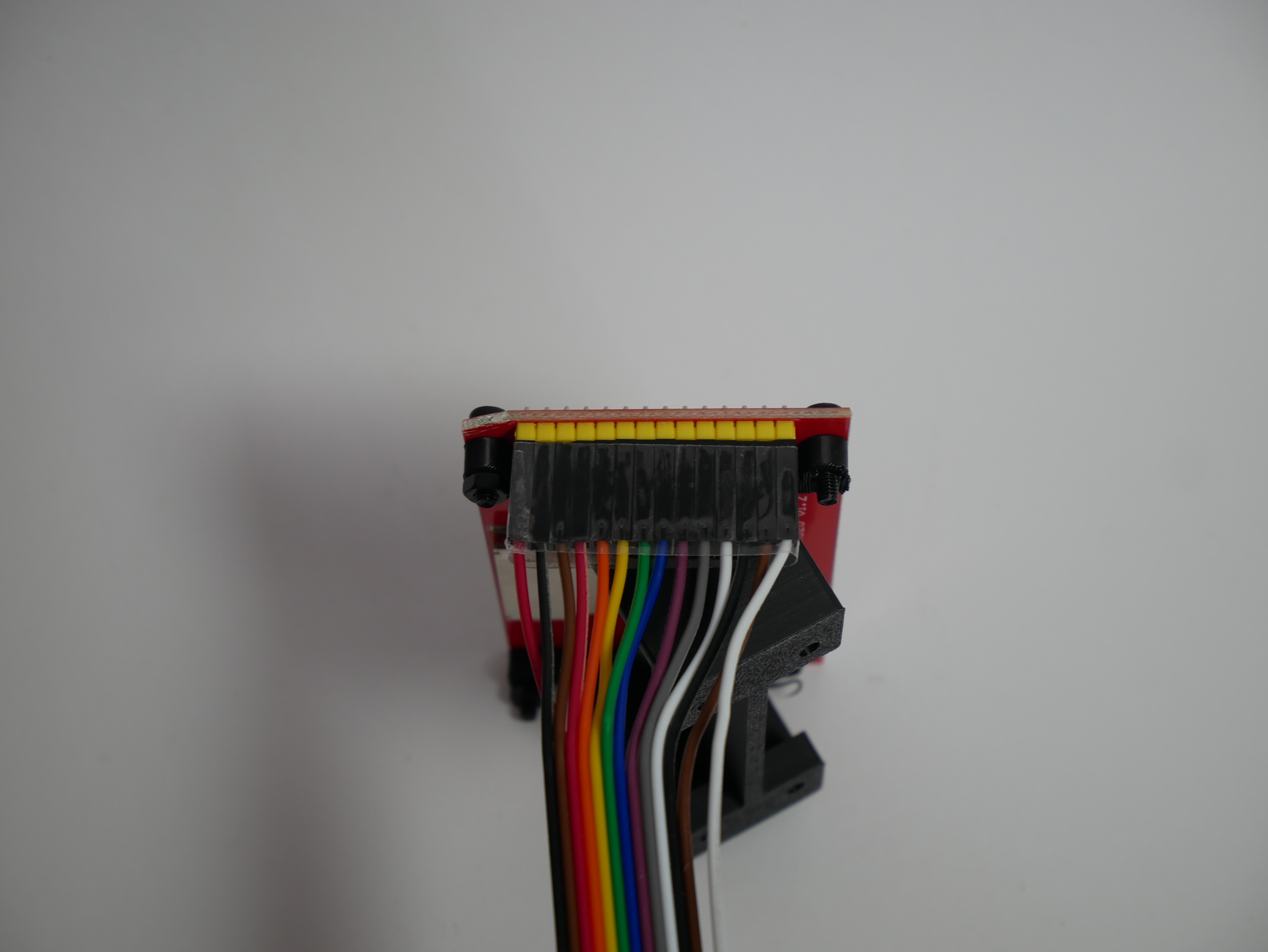

Supplement: Supplementary file 1 — Supplementary materials [file 41378_2024_856_MOESM1_ESM.zip › Supplementary Materials/Figures/display_cables.JPG]

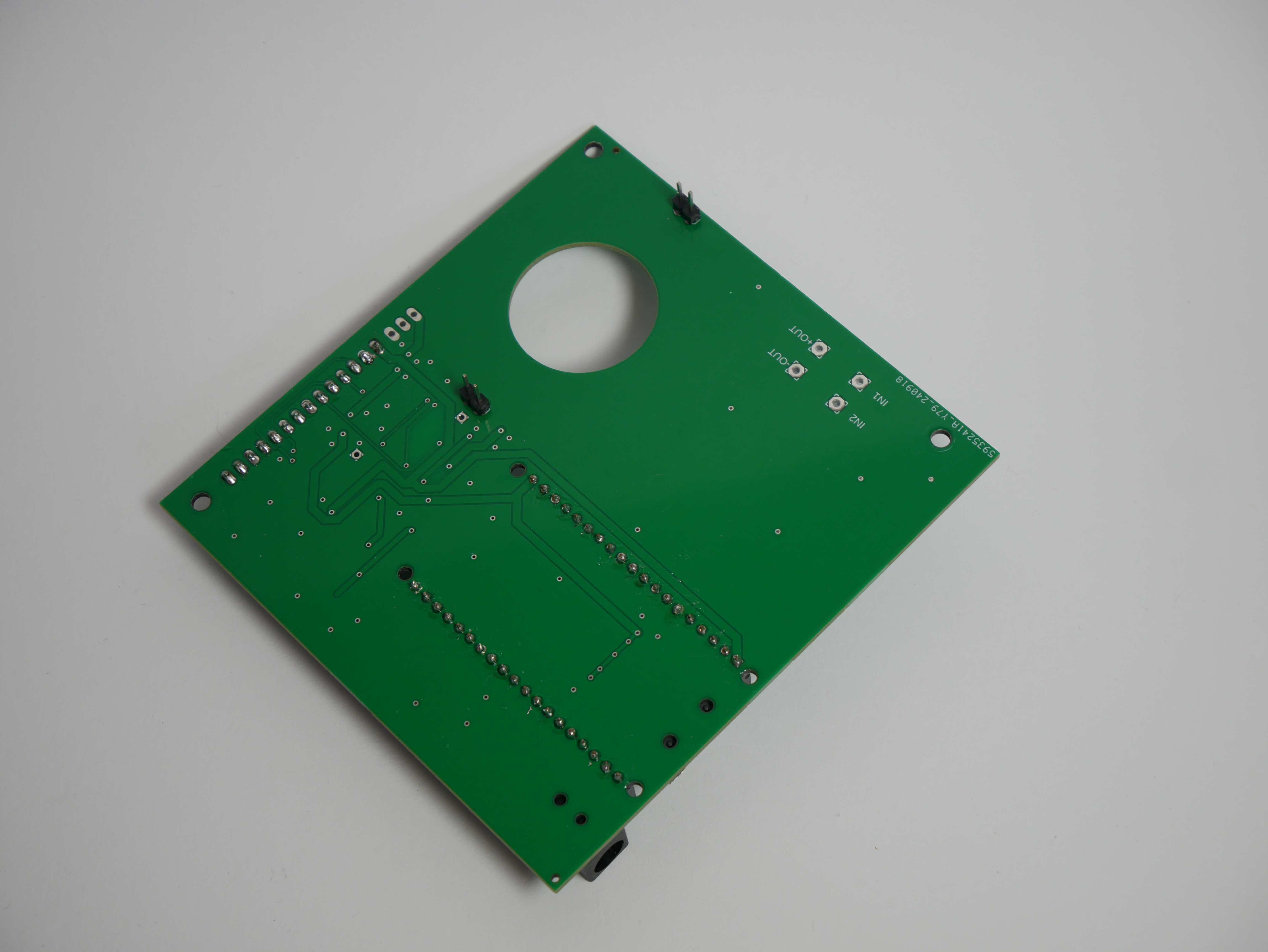

Supplement: Supplementary file 1 — Supplementary materials [file 41378_2024_856_MOESM1_ESM.zip › Supplementary Materials/Figures/headers_bottom.JPG]

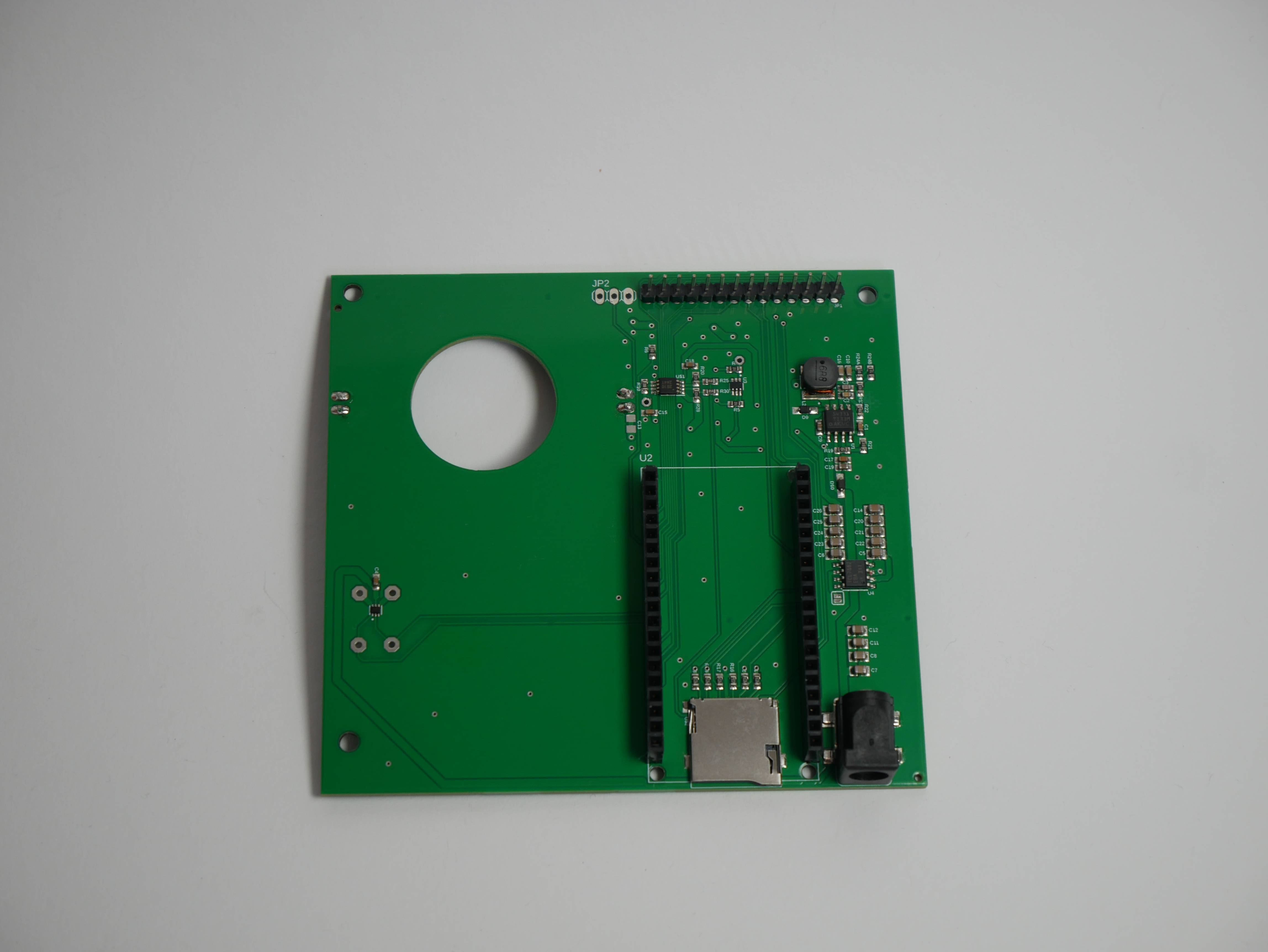

Supplement: Supplementary file 1 — Supplementary materials [file 41378_2024_856_MOESM1_ESM.zip › Supplementary Materials/Figures/headers_top.JPG]

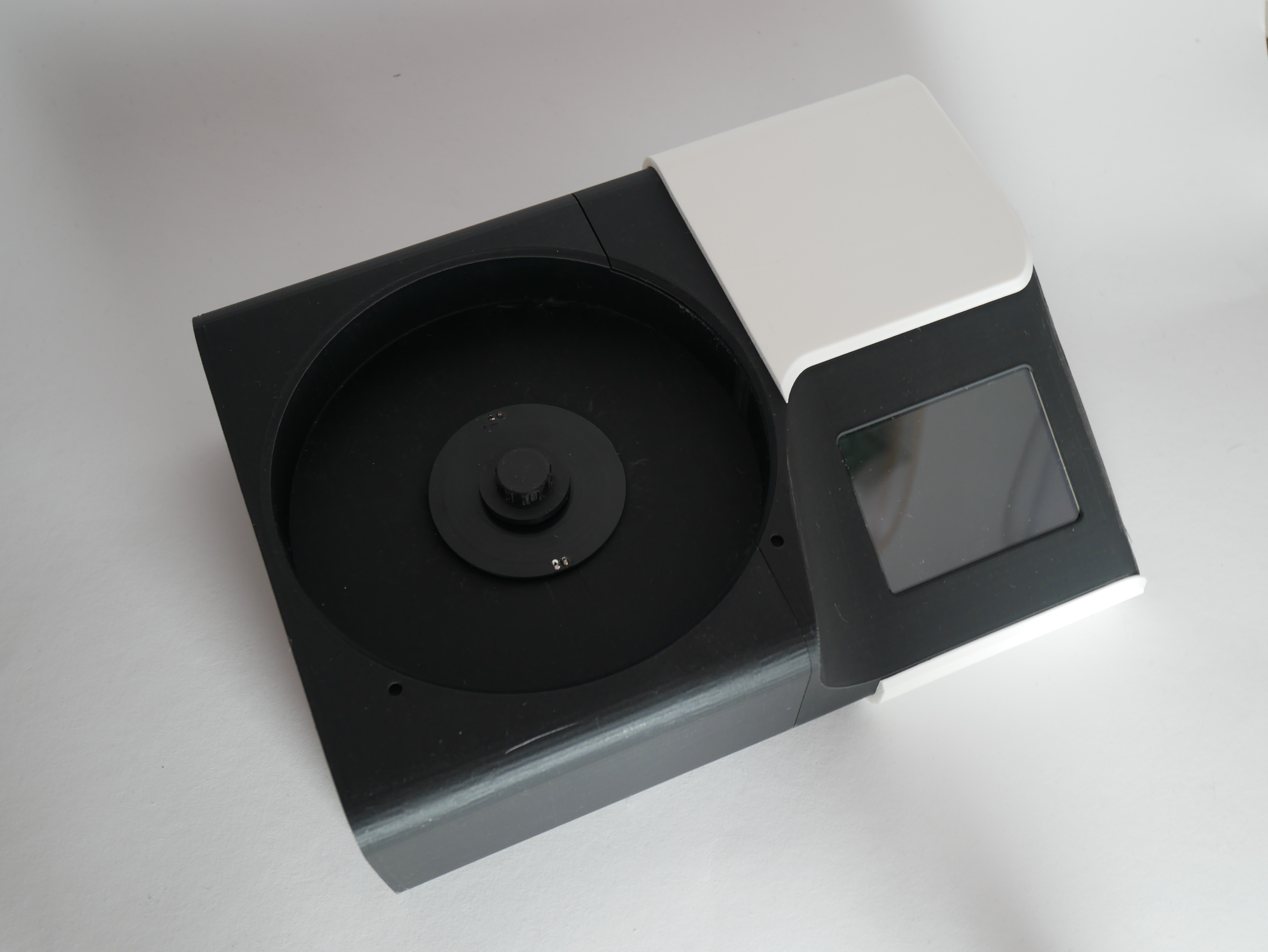

Supplement: Supplementary file 1 — Supplementary materials [file 41378_2024_856_MOESM1_ESM.zip › Supplementary Materials/Figures/right_outer.JPG]

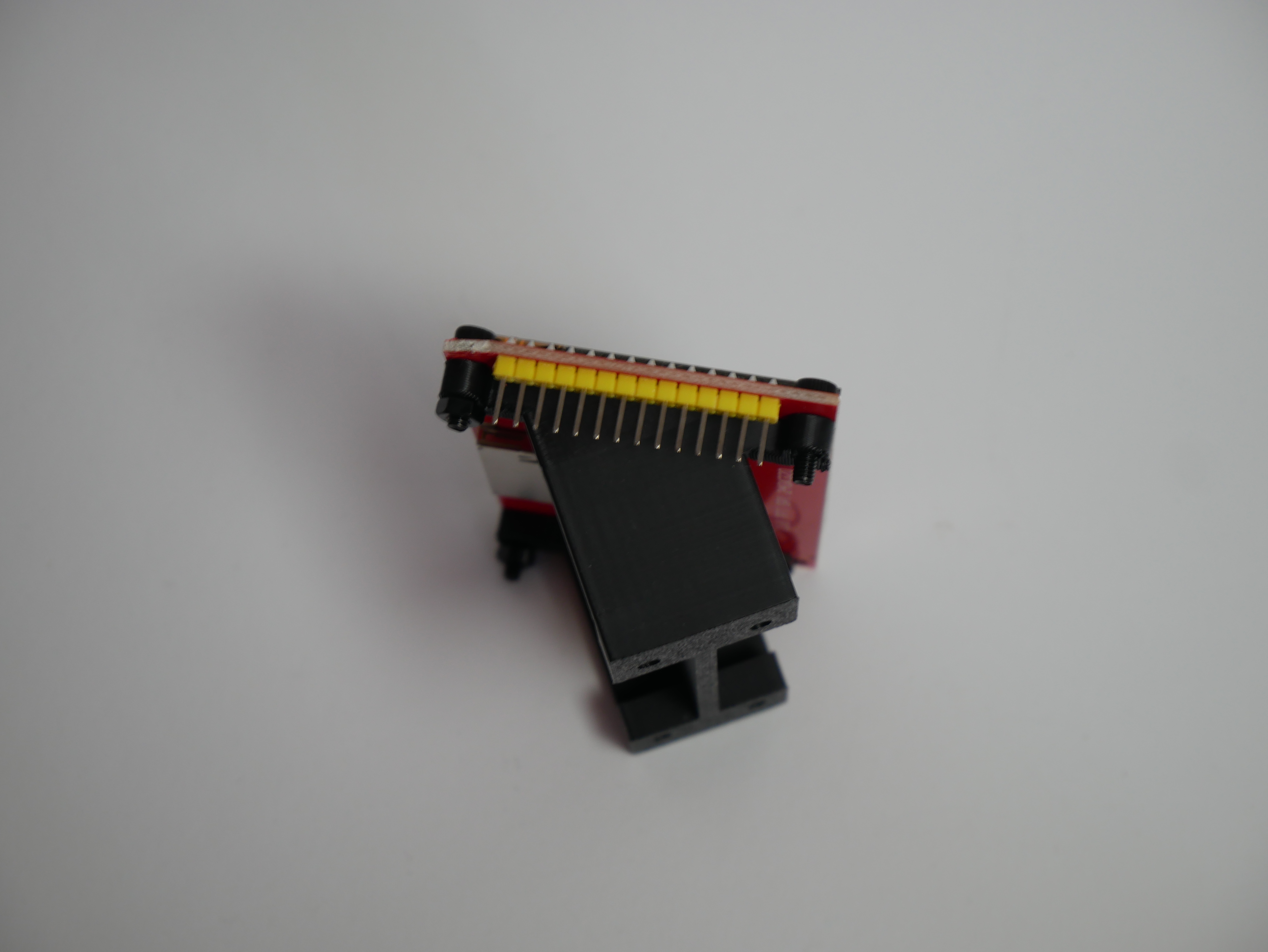

Supplement: Supplementary file 1 — Supplementary materials [file 41378_2024_856_MOESM1_ESM.zip › Supplementary Materials/Figures/display_mount.JPG]
